# Targeting IL13Ralpha2 activates STAT6-TP63 pathway to suppress breast cancer lung metastasis

**DOI:** 10.1186/s13058-015-0607-y

**Published:** 2015-07-25

**Authors:** Panagiotis Papageorgis, Sait Ozturk, Arthur W. Lambert, Christiana M. Neophytou, Alexandros Tzatsos, Chen K. Wong, Sam Thiagalingam, Andreas I. Constantinou

**Affiliations:** Department of Biological Sciences, School of Pure and Applied Sciences, University of Cyprus, 75 Kallipoleos Ave., 1678 Nicosia, Cyprus; Departments of Medicine (Biomedical Genetics Section, Molecular Medicine Program and Cancer Center), Genetics & Genomics and Pathology & Laboratory Medicine, Boston University School of Medicine, 72 East Concord Street, Boston, MA 02118 USA; Department of Health Sciences, European University Cyprus, 6 Diogenis Street, 2404 Nicosia, Cyprus; Department of Anatomy and Regenerative Biology, George Washington University School of Medicine, 2300 I Street NW, Washington, DC 20037 USA

## Abstract

**Introduction:**

Basal-like breast cancer (BLBC) is an aggressive subtype often characterized by distant metastasis, poor patient prognosis, and limited treatment options. Therefore, the discovery of alternative targets to restrain its metastatic potential is urgently needed. In this study, we aimed to identify novel genes that drive metastasis of BLBC and to elucidate the underlying mechanisms of action.

**Methods:**

An unbiased approach using gene expression profiling of a BLBC progression model and *in silico* leveraging of pre-existing tumor transcriptomes were used to uncover metastasis-promoting genes. Lentiviral-mediated knockdown of interleukin-13 receptor alpha 2 (IL13Ralpha2) coupled with whole-body in vivo bioluminescence imaging was performed to assess its role in regulating breast cancer tumor growth and lung metastasis. Gene expression microarray analysis was followed by in vitro validation and cell migration assays to elucidate the downstream molecular pathways involved in this process.

**Results:**

We found that overexpression of the decoy receptor IL13Ralpha2 is significantly enriched in basal compared with luminal primary breast tumors as well as in a subset of metastatic basal-B breast cancer cells. Importantly, breast cancer patients with high-grade tumors and increased IL13Ralpha2 levels had significantly worse prognosis for metastasis-free survival compared with patients with low expression. Depletion of IL13Ralpha2 in metastatic breast cancer cells modestly delayed primary tumor growth but dramatically suppressed lung metastasis in vivo. Furthermore, IL13Ralpha2 silencing was associated with enhanced IL-13-mediated phosphorylation of signal transducer and activator of transcription 6 (STAT6) and impaired migratory ability of metastatic breast cancer cells. Interestingly, genome-wide transcriptional analysis revealed that IL13Ralpha2 knockdown and IL-13 treatment cooperatively upregulated the metastasis suppressor tumor protein 63 (TP63) in a STAT6-dependent manner. These observations are consistent with increased metastasis-free survival of breast cancer patients with high levels of *TP63* and *STAT6* expression and suggest that the STAT6-TP63 pathway could be involved in impairing metastatic dissemination of breast cancer cells to the lungs.

**Conclusion:**

Our findings indicate that IL13Ralpha2 could be used as a promising biomarker to predict patient outcome and provide a rationale for assessing the efficacy of anti-IL13Ralpha2 therapies in a subset of highly aggressive basal-like breast tumors as a strategy to prevent metastatic disease.

**Electronic supplementary material:**

The online version of this article (doi:10.1186/s13058-015-0607-y) contains supplementary material, which is available to authorized users.

## Introduction

It is widely accepted that metastasis accounts for the vast majority of deaths of patients with breast cancer. Cancer metastasis represents a multistep event which can be broadly divided in two phases: The first one represents the physical dissemination of cancer cells to distant tissues, and the second involves the development of macrometastatic lesions in these organs [1]. These steps involve the sequential acquisition of genetic and epigenetic alterations which provide a level of cellular plasticity that is indispensable for the completion of the metastatic process [2, 3]. It is also clear that complex tumor-host cell interactions are critically important for the adaptation of cancer cells in a foreign tissue microenvironment, which fosters macroscopic colonization [4].

Interleukins (IL) comprise a superfamily of pleiotropically acting cytokines that are present in the tumor microenvironment and are implicated in a wide variety of immunomodulatory functions, including cell maturation, proliferation, migration, and adhesion [5]. In addition to cells of the immune system, it is becoming increasingly clear that these cytokines can exert their effects on epithelial cancer cells in an autocrine or paracrine fashion, thereby regulating tumorigenesis and cancer metastasis [6–8]. Thus, the expression patterns of cytokine receptors on cancer cells may alter their response to the tumor microenvironment and influence their tumorigenic and metastatic potential. IL-13 is a T helper 2 (Th2) cell-derived cytokine that plays a pivotal role in inflammation and immune system regulation [9]. It shares structural and biological similarities with IL-4 and is known to function by initially binding to IL13Rα1, followed by recruitment and heterodimerization with IL4Rα to transduce signaling predominantly via the Janus kinase 2-signal transducer and activator of transcription (JAK-STAT) pathway [10–12]. On the other hand, IL13Rα2 is a high-affinity receptor for binding and internalization of IL-13 which is unable to transduce downstream signaling potentially because of its short cytoplasmic domain [13]. Thus, IL13Rα2 can function as a decoy receptor by competing with IL13Rα1 for ligand binding in order to inhibit downstream STAT signaling and IL-13 responses [14–17]. Interestingly, high IL13Rα2 expression levels have been associated with the development of gliomas as well as head and neck cancers [18, 19] and were also shown to promote invasion and metastasis of pancreatic, ovarian, and colorectal cancers [20–22]. Although a previous gene expression profiling study indicated that IL13Rα2 is overexpressed in breast tumors from patients who developed lung metastases [23], its functional role and underlying mechanism of action in breast cancer development and progression remain largely unknown.

Despite much effort in recent years, the discovery of critical mediators of breast cancer metastasis which could also represent feasible targets for therapy is still a major challenge, particularly for the aggressive basal-like subtype tumors, the majority of which are classified as triple-negative: estrogen receptor-/progesterone receptor-/human epidermal growth factor receptor 2-negative (ER^−^/PR^−^/Her2^−^). Here, using unbiased gene expression profiling of a well-described human basal-like breast cancer (BLBC) model system, we identified IL13Rα2 as a potent driver of breast cancer metastasis. We found high expression levels of this receptor to be associated with basal compared with luminal primary breast tumors and enriched in a subset of metastatic basal-B breast cancer cells. Increased *IL13Rα2* levels were also associated with poor metastasis-free survival of patients with breast cancer. Importantly, targeted depletion of IL13Rα2 resulted in dramatic suppression of lung metastasis formation in vivo that is likely to be attributed, at least in part, to STAT6-dependent induction of tumor protein 63 (TP63) expression and suppression of breast cancer cell migration.

## Methods

### Cell culture

MCF10A (MI), MCF10ATk1.cl2 (MII), MCF10CA1h (MIII), and MCF10CA1a (MIV) breast cancer cell lines were obtained from the Karmanos Cancer Institute (Detroit, MI, USA) and maintained as previously described [2]. SUM159, SKBR3, MDA-MB-361 cells were a kind gift from Ramon Parsons, of Mount Sinai Hospital (New York, NY, USA), and MDA-MB-231-LM2 cells were a kind gift from Joan Massague, of Memorial Sloan Kettering Cancer Center (MSKCC) (New York, NY, USA). MDA-MB-231 cells were purchased from ATCC (Manassas, VA, USA). These cells were maintained in Dulbecco’s modified Eagle’s medium (DMEM) supplemented with 10 % fetal bovine serum (FBS).

### Antibodies and reagents

Antibodies were purchased from the following sources: goat anti-IL13Rα2 (R&D Systems, Minneapolis, MN, USA), mouse anti-IL13Rα2, mouse anti-α-tubulin, and mouse anti-GAPDH (Santa Cruz Biotechnology, Inc., Dallas, TX, USA), STAT6, P-STAT1, P-STAT3, P-STAT5, P-STAT6 (S737), P-STAT6 (Y641), mouse anti-HA (Roche, Basel, Switzerland), and anti-TP63α (Cell Signaling Technology, Beverly, MA, USA). PCMV6 empty vector or pCMV6-IL13Rα2-Myc-Flag constructs were purchased from OriGene (Rockville, MD, USA).

### RNA isolation, cDNA synthesis, and real-time polymerase chain reaction

Total RNA was isolated by using Trizol (Invitrogen, part of Thermo Fisher Scientific, Waltham, MA, USA), and cDNA synthesis was performed by using RT-III enzyme and random hexamers (Invitrogen). Real-time polymerase chain reaction (PCR) was performed by using primers listed in Additional file 1. All protocols were previously described [2].

### Western blotting

Whole protein cell lysates were isolated by using radioimmunoprecipitation assay buffer containing protease and phosphatase inhibitors. Sodium dodecyl sulfate-polyacrylamide gel electrophoresis (SDS-PAGE) analysis was performed as previously described [2]. All Western blot experiments were independently performed at least three times, and the representative images shown were quantified by using ImageJ software.

### Kaplan-Meier plotter analysis

Kaplan-Meier plotter [24], an *in silico* online tool, was used to predict survival of breast cancer patients on the basis of expression of candidate genes. Affymetrix gene expression data from multiple annotated breast cancer studies are combined into a single database from which we queried for associations between expression of selected genes and patient outcome [25].

### Meta-analysis of the MSKCC primary breast tumor cohort

Associations between IL13Rα2 expression levels and ER or PR or Her2 receptor or basal and luminal breast tumor subtypes or patient prognosis were investigated by performing meta-analysis of the MSKCC primary breast tumor cohort, as previously published [23]. Breast tumor subtypes were determined on the basis of the expression of keratin 5 and keratin 17 (basal) or keratin 8 and keratin 18 (luminal) markers [26]. Tumors were stratified on the basis of the median IL13Rα2 expression levels in low- or high-expression groups. Chi-squared analysis was performed to reveal associations between the different above-mentioned tumor characteristics.

### Cloning of small hairpin RNA-expressing vectors and viral transduction

To generate lentiviral vectors expressing small hairpin RNA (shRNAs) against *IL13Rα2* and *STAT6*, we used AgeI/EcoRI-digested pLKO.1-puro vector ligated with 58 base pair-oligos (listed in Additional files 1) [27]. Establishment of MIV and SUM159 cells stably expressing different shRNA constructs was performed by lentiviral-mediated transduction. Briefly, 293T cells were co-transfected with 5 μg pLKO-shScrambled or pLKO-shIL13Rα2 #1 or #2 or pLKO-shSTAT6 #1 or #2 with 3 μg psPAX2 and 1 μg pMD2.G plasmids. After 48 h, MIV or SUM159 cells were transduced with virus-containing medium in the presence of 10 μg/ml polybrene, selected with 2 μg/ml puromycin, and pooled for further assays. MIV cells stably expressing the luciferase gene (MIV-Luc) were generated by co-transfection of pMSCV-Luc-PGK-hygro retroviral vector (gift from Scott Lowe, Addgene-8782) (4 μg) with pCL1-ampho packaging plasmid (4 μg) in 293T cells. Viral supernatant was used for transduction of target cells by using polybrene. Transduced cells were selected by using 100 μg/ml hygromycin, and resistant cells were pooled for further assays.

### Transient transfection assays

Transient transfection assays were performed by transfecting MCF10A (MI) cells with 3 μg pCMV6 empty vector or pCMV6-shIL13Rα2-Myc-Flag construct (OriGene) along with 9 μl of X-tremeGene 9 transfection reagent (Roche). Cells were allowed to grow for 48 h before various treatments with IL-13.

### Transwell migration assays

Chemotaxis migration assays were performed by using six-well transwell plates containing 8.0 μm-pore membrane (Corning, Corning, NY, USA). Serum-free DMEM/F-12 (1 ml-control) or DMEM/F-12 +10 % FBS medium (1 ml) was added in the bottom chamber, and 3×10^5^ MIV-Luc-shSCR cells or MIV-Luc-shIL13Rα2#2 cells were resuspended in 1 ml of serum-free DMEM/F-12 and plated on the upper insert membrane. Cells were then treated with or without 20 ng/ml IL-13 for 48 h, fixed with methanol, and stained with trypan blue (0.4 %). Migrated cells localized on the bottom membrane surface were imaged and counted by using an Axiovert 200M inverted microscope (Carl Zeiss, Oberkochen, Germany) (10× magnification, at least five fields per condition).

### Tumorigenesis and metastasis assays coupled with whole-body in vivo bioluminescence imaging

In vivo studies were conducted at the facilities of Boston University School of Medicine under animal protocol AN-14844 approved by the Institutional Animal Care and Use Committee. Tumorigenesis assays were performed by subcutaneously injecting 6-week-old female NOD.CB17-*Prkdc*^*scid*^/J mice (The Jackson Laboratory, Bar Harbor, ME, USA) with 5×10^5^ MIV-Luc-shSCR or MIV-Luc-shIL13Rα2#2 cells suspended in 0.1 ml of serum-free DMEM/F-12 medium. For metastasis assays, mice were injected with 5×10^5^ MIV-Luc-shSCR or MIV-Luc-shIL13Rα2#2 cells via the lateral tail vein. The growth rate of tumors in animal tissues was monitored once a week by using whole-body in vivo bioluminescence imaging. Mice were anesthetized by inhalation of 2 % isofluorane, and D-luciferin (150 mg/kg) was injected intraperitoneally 30 min prior to measurements. The rate of photon flux (photons/square cm per sec) was quantified by using an IVIS Spectrum imaging system (PerkinElmer, Waltham, MA, USA) and the Living Image software. Mice bearing primary tumors were euthanized when tumors reached approximately 1 cm in diameter or became ulcerated. Mice developing lung metastases were euthanized when they developed cachexia symptoms. Primary tumors and murine lungs were excised for measurement of tumor weight and assessment of lung metastasis formation, respectively. In vivo tumorigenesis assay was performed four independent times, whereas metastasis assays were independently performed twice.

### Histological analysis and immunohistochemistry

Primary tumors or lungs were isolated from mice injected with either MIV-Luc-shSCR or MIV-Luc-shIL13Rα2#2 breast cancer cells, fixed in 4 % parafolmaldehyde, and embedded in paraffin. Tissue sections (10 μm thick) were performed by using a Leica RM2125RT microtome (Leica Biosystems, Nussloch, Germany), followed by staining with hematoxylin and eosin (H&E) by using standard methodology. Immunohistochemical detection of mitotic cells and IL13Rα2 expression were generated by staining sections with anti-Ki67 antibody (clone MIB1) and goat anti-IL13Rα2 (R&D Systems), respectively, followed by horseradish peroxidase-conjugated secondary antibodies and counterstained with H&E. Bright-field images of stained slides were obtained by using an Axiovert 200M inverted microscope.

### Microarray gene expression analysis

To identify downstream effectors of IL13Rα2, four biological conditions were used: (1) MIV-Luc-shSCR cells mock-treated (− IL-13), (2) MIV-Luc-shSCR cells treated with 20 ng/ml IL-13 for 16 h (+ IL-13), (3) MIV-Luc-shIL13Rα2#2 (− IL-13), and (4) MIV-Luc-shIL13Rα2#2 (+ IL-13). Total RNA was isolated from two biological replicates of each condition by using an RNeasy mini-kit (Qiagen, Hilden, Germany). Hybridization and initial analysis were performed by ATLAS Biolabs GmbH (Berlin, Germany) by using GeneChip Human Exon 1.0ST arrays (Affymetrix, Santa Clara, CA, USA). Raw data quality control and normalization were performed by using the Affymetrix Expression Console, whereas differentially expressed genes were determined with the Affymetrix Transcriptome Analysis Console (one-way analysis of variance *P* value of less than 0.05, fold change of more than or less than 2, false discovery rate of less than 0.05). Hierarchical clustering was performed with the TreeView Software. The microarray data generated are available from the National Center for Biotechnology Information (NCBI) Gene Expression Omnibus [28] under accession code GSE57677.

### Statistical analysis

Two-tailed unpaired Student’s *t* test was performed for statistical analysis of real-time PCR, migration assays, and Western blot analyses. For in vivo experiments, two-tailed unpaired Student’s *t* test or Mann-Whitney test was performed to assess statistical significance. All data are presented as mean ± standard error and are representative of at least three independent experiments, unless stated otherwise. Chi-squared analysis was performed to investigate associations between IL13Rα2 expression and various primary breast tumor features of the MSKCC cohort. *P* values of less than 0.05 were considered statistically significant between the compared samples and are indicated by the (*) symbol for *P* < 0.05, by (**) for *P* < 0.01, or by (***) for *P* < 0.001 in respective figures.

## Results

### IL13Rα2 is overexpressed in metastatic breast cancer cells and is associated with poor prognosis for metastasis-free survival of patients with breast cancer

**Fig. 1:**
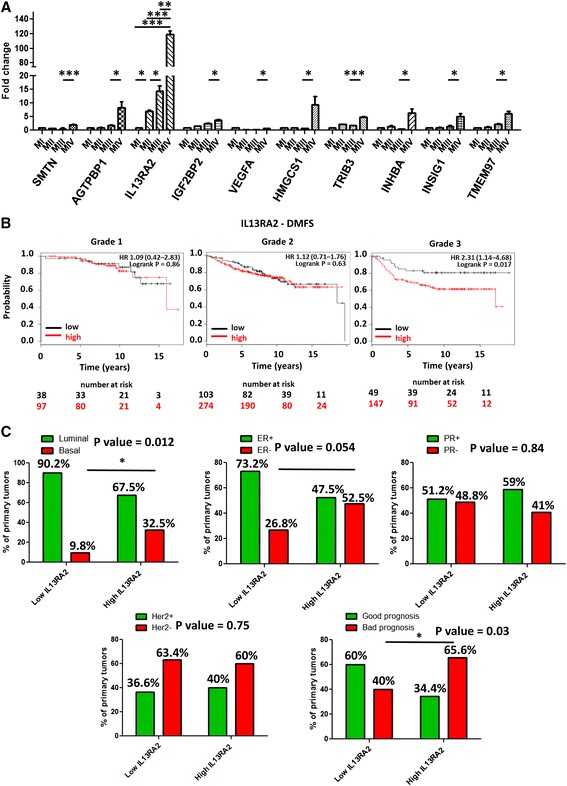
IL13Rα2 is overexpressed in metastatic basal-like breast cancers and is associated with poor survival. **a** Real-time polymerase chain reaction analysis of the candidate metastasis-promoting genes *SMTN*, *AGTPBP1*, *IL13Rα2*, *IGF2BP2*, *VEGFA*, *HMGCS1*, *TRIB3*, *INHBA*, *INSIG1*, and *TMEM97* to examine their expression levels in the MI, MII, MIII, and MIV cells. *P < 0.05, **P < 0.01, ***P < 0.001. **b** Kaplan-Meier survival analysis for assessment of distant metastasis-free survival (*DMFS*) based on tumor *IL13RΑ2* expression in 135 patients with grade 1, 377 patients with grade 2, and 196 patients with grade 3 breast cancer [25]. Survival curves were generated by using the Kaplan-Meier Plotter online tool based on data stratified at the lower quartile (lowest 25 % *IL13Rα2* expression versus all others). Curves were compared by log-rank test. **c** Meta-analysis of the Memorial Sloan Kettering Cancer Center primary breast tumor cohort [23] was performed to identify associations between *IL13Rα2* expression levels and basal versus luminal breast tumor subtypes; estrogen receptor (*ER*), progesterone receptor (*PR*), or human epidermal growth factor 2 (*Her2*) receptor status; and patient prognosis. Tumors were stratified on the basis of the median IL13Rα2 expression levels in low- or high-expression groups. Breast tumor subtypes were determined on the basis of the expression of keratin 5 and keratin 17 (basal) or keratin 8 and keratin 18 (luminal) markers [26]. Statistical significance was assessed by using chi-squared analysis. *P < 0.05. *IL13Rα2* interleukin-13 receptor alpha 2

The lack of promising molecular targets against breast cancer metastasis led us to design a strategy for the identification of novel metastasis-promoting genes. To this end, we exploited a well-established cell line model system for breast cancer progression which is transcriptionally classified under the basal-B subtype [2, 29]. It consists of the MCF10A (MI) spontaneously immortalized mammary epithelial cell line and three of its derivatives, namely MII, MIII, and MIV, obtained after serial passaging in nude mice [30]. These cell lines exhibit distinct tumorigenic and metastatic properties when re-implanted in immunodeficient mice; MI is non-tumorigenic, MII forms benign hyperplastic lesions, MIII forms low-grade, well-differentiated carcinomas, whereas MIV develops high-grade, poorly differentiated metastatic carcinomas [31]. To identify candidate genes that promote breast cancer metastasis, we re-analyzed our recently generated gene expression microarray data from this cell line system [32]. We focused on cluster 9 containing 29 genes significantly upregulated in the metastatic MIV compared with their non-metastatic counterparts, the MII and MIII cell lines (Additional file 2: Figure S1). This list of genes was filtered by using an online tool which performs meta-analysis of publicly available microarray datasets from patients with breast cancer to generate Kaplan-Meier survival curves [25]. During this analysis, patients were separated into two groups on the basis of the expression levels of each gene, and the probability of metastasis-free survival over time was calculated. We found that higher expression of 10 out of the 29 genes could individually predict worse distant metastasis-free survival (DMFS) of patients with breast cancer (Additional file 3: Figure S2). The expression pattern of these genes was then validated in MI-MIV cells by using real-time PCR. Interestingly, *IL13Rα2* was the most highly upregulated gene in metastatic MIV cells compared with its non-metastatic counterparts (Fig. 1a). In addition, high *IL13Rα2* expression could specifically predict metastasis-free survival of patients with high- but not low-grade tumors (Fig. 1b), suggesting that overexpression of this gene may be involved in promoting the late stages of tumor progression. To further support these findings, we performed a meta-analysis of gene expression microarray data from the MSKCC primary breast tumor cohort [23] which indicated that high *IL13Rα2* levels are significantly associated with the basal compared with luminal tumor subtype as well as bad prognosis. Increased *IL13Rα2* expression was also marginally associated with ER^−^ breast tumors, whereas no association was found with PR or Her2 status (Fig. 1c). We also performed a meta-analysis of two additional published microarray datasets from breast cancer cell lines of various subtypes [29] as well as from metastatic variants of MDA-MB-231 breast cancer cells [23]. Based on this analysis as well as on validation in a subset of these cell lines by real-time PCR and Western blotting, we found that *IL13Rα2* overexpression is exclusively enriched in a subset of basal-B breast cancer cells that are highly metastatic to the lungs (MCF10CA1a, MDA-MB-436, BT549, SUM159, and MDA-MB-231-LM2-4175) [23, 31, 33, 34] but not in any of the luminal or basal-A subtype cells (Additional file 4: Figure S3 and Additional file 5: Figure S4). Collectively, these data indicated that high *IL13Rα2* levels might be involved in metastatic progression of basal-like breast tumors.

### IL13Rα2 depletion enhances IL-13-mediated STAT6 phosphorylation

**Fig. 2:**
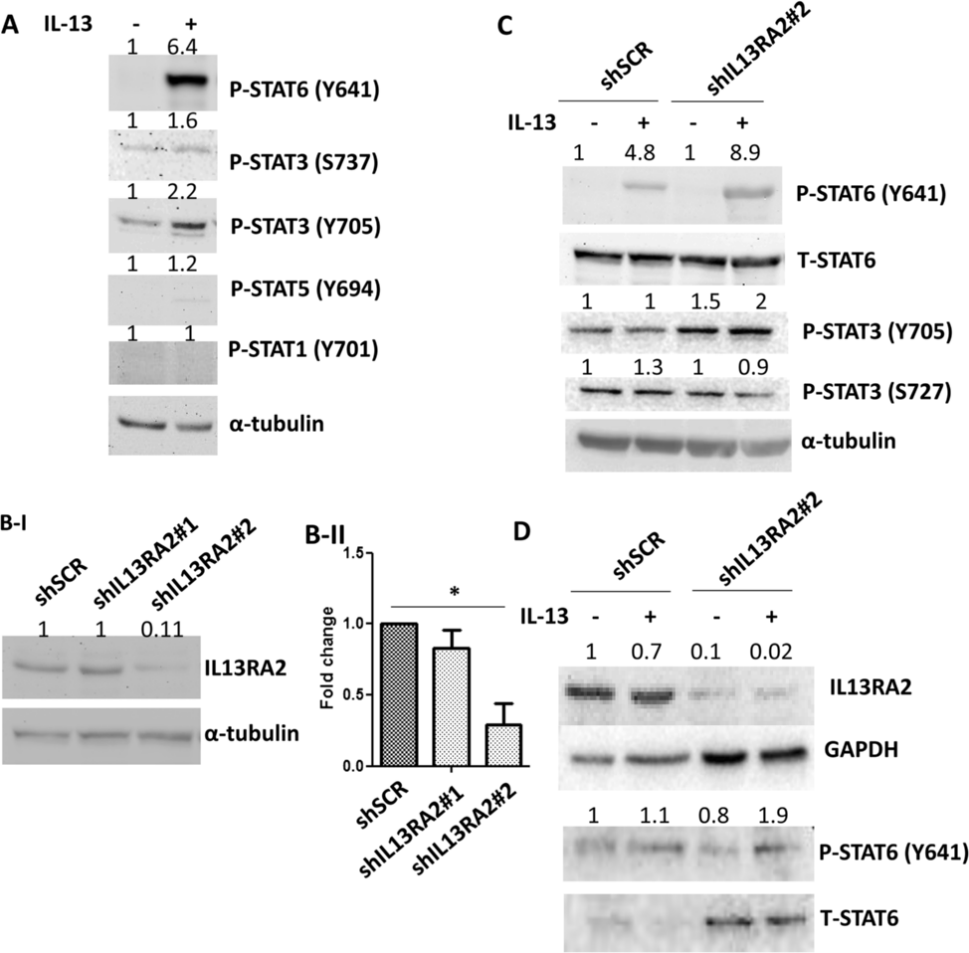
Knockdown of IL13Rα2 enhances IL-13-mediated STAT6 phosphorylation. **a** MIV cells were treated with 20 ng/ml IL-13 for 30 min, and Western blot analysis was performed with whole cell lysates to assess phosphorylation levels of STAT1, STAT3, STAT5, and STAT6. **b** Western blot (i) and real-time polymerase chain reaction (ii) were performed in MIV cells stably transduced with shSCR or shIL13Rα2 lentiviral vectors expressing two different small hairpin RNA oligos (#1 and #2) to measure shIL13Rα2 protein and mRNA levels, respectively. **c** MIV-shSCR and MIV-shIL13Rα2#2 cells were treated with 1 ng/ml IL-13 for 30 min, and Western blot analysis was performed with whole cell lysates to assess phosphorylation levels of STAT6 (Y641) and STAT3 (Y705 and S727). Total STAT6 and α-tubulin protein levels were detected as loading controls. **d** SUM159-shSCR and SUM159- shIL13Rα2#2 cells were treated with 1 ng/ml IL-13 for 30 min, and Western blot analysis was performed with whole cell lysates to assess phosphorylation levels of STAT6 (Y641). Total STAT6 and GAPDH protein levels were detected as loading controls. *IL*-*13* interleukin-13, *IL13Rα2* interleukin-13 receptor alpha 2, *shIL13Rα2* small hairpin RNA against interleukin-13 receptor alpha 2, *shSCR* scrambled small hairpin RNA, *STAT* signal transducer and activator of transcription

It is well established that IL-13 can induce the phosphorylation and activation of various STAT protein family members, in a context-dependent manner, to regulate diverse cellular properties [35]. To investigate which is the predominant STAT protein that is activated by IL-13 in our system, we treated MIV cells with IL-13 and analyzed the activation of STAT proteins. We found that STAT6 was robustly phosphorylated at Tyr641 by IL-13 but that STAT3 phosphorylation at Tyr705 appeared to be only modestly induced. In contrast, STAT3 (Ser737), STAT5 (Tyr694), and STAT1 (Tyr701) residues were not affected by IL-13 treatment (Fig. 2a). Because IL13Rα2 acts as a negative regulator of the IL-13 pathway, we wanted to assess whether IL13Rα2 overexpression can aberrantly regulate IL-13-mediated STAT signaling by predominantly targeting STAT6. To obtain further insights, we first confirmed that the IL13Rα2 protein and mRNA are overexpressed in metastatic MIV compared with non-metastatic MI, MII, and MIII cells (Additional file 5: Figure S4a, S4b). Then, we constructed lentiviral vectors expressing shRNA against IL13Rα2 and stably knocked down *IL13Rα2* expression in metastatic MIV cells (Fig. 2b). Treatment of MIV-shSCR or MIV-shIL13Rα2#2 cells with IL-13 followed by Western blotting analysis revealed that, upon *IL13Rα2* knockdown, there was a significant increase in the level of STAT6 phosphorylation mediated by IL-13 (Fig. 2c). To verify that this effect is not cell line-specific, we also stably depleted *IL13Rα2* in SUM159 cells. Comparison between SUM159-shSCR and SUM159-shIL13Rα2 cells indicated that there was a similar enhancement of STAT6 activation by IL-13 in the absence of IL13Rα2 (Fig. 2d). In addition, we performed the reverse experiment by overexpressing IL13Rα2 in the non-tumorigenic MCF10A (MI) cell line which resulted in inhibition of STAT6 activation by IL-13 (Additional file 6: Figure S5a, S5b). Overall, these data demonstrate that high levels of IL13Rα2 can suppress IL-13-mediated STAT6 phosphorylation.

### Knockdown of IL13Rα2 modestly delays primary breast tumor growth and suppresses breast cancer metastasis to the lungs

**Fig. 3:**
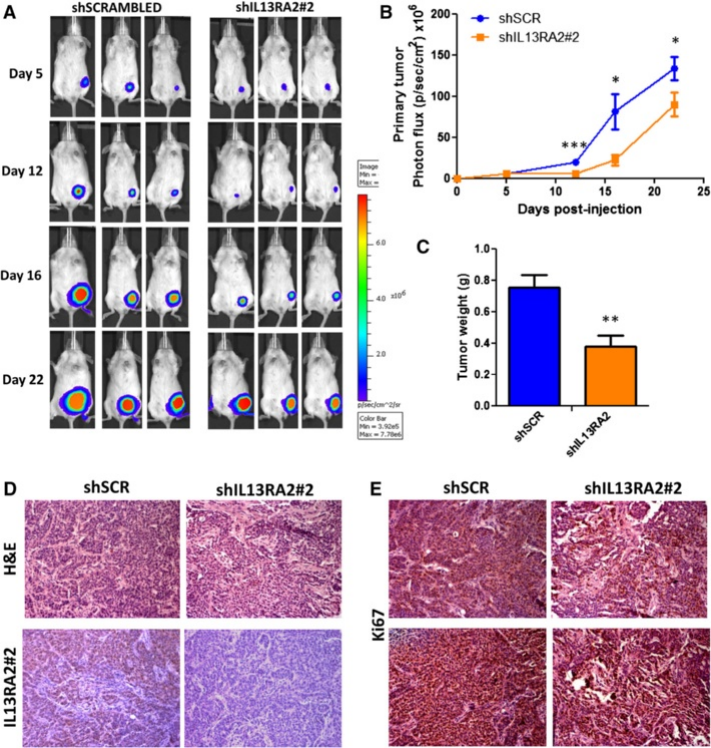
IL13Rα2 depletion delays growth of primary breast tumors. **a** Representative in vivo bioluminescence images of non-obese diabetic/severe combined immunodeficient mice injected subcutaneously with 5×10^5^ MIV-Luc-shSCR or MIV-Luc-shIL13Rα2#2 cells at different time points post-implantation. **b** Quantification and comparison of photon flux (photons/sec per cm^2^) over time (Student’s *t* test, day 5 *P* value = 0.96, day 12 *P* value = 0.0003, day 16 *P* value = 0.017 and day 22 *P* value = 0.042, *n* = 11). *P < 0.05, **P < 0.01, ***P < 0.001. **c** Comparison of primary tumor weight between MIV-Luc-shSCR and MIV-Luc-shIL13Rα2#2 cell-derived tumors at the experimental endpoint 22 days post-implantation (Student’s *t* test, *P* value = 0.0017, *n* = 15). **P < 0.01. Representative images (**d**) from hematoxylin and eosin (*H&E*), anti-IL13Rα2 (brown) and (**e**) from anti-Ki67 staining (brown) of 10 μm-thick sections from paraffin-embedded primary tumors isolated from mice injected with MIV-Luc-shSCR or MIV-Luc-shIL13Rα2#2 cells (20×). *IL13Rα2* interleukin-13 receptor alpha 2, *shIL13Rα2* small hairpin RNA against interleukin-13 receptor alpha 2, *shSCR* scrambled small hairpin RNA

Because we found that targeting IL13Rα2 enhances STAT6 phosphorylation by IL-13, we hypothesized that this induction may be implicated in modulating the tumorigenic and metastatic properties of breast cancer cells. To address this question, we stably introduced a luciferase reporter in MIV cells to monitor and quantify the spatiotemporal growth of tumors, generated upon injection of cells in immunodeficient mice, by non-invasive whole-body bioluminescence imaging. First, we performed in vivo tumorigenesis assays to compare the effect of IL13Rα2 expression on the tumorigenic potential of MIV cells. MIV-Luc cells stably transduced with either scrambled shRNA (MIV-Luc-shSCR) or shRNA against *IL13Rα2* (MIV-Luc-shIL13Rα2#2 cells) were subcutaneously injected in non-obese diabetic/severe combined immunodeficient (NOD/SCID) mice. We found that there was a delay in the tumor growth of IL13Rα2-depleted MIV-Luc cells compared with controls and that this was attributed predominantly to a significant, albeit modest, reduction during days 12–16 post-injection (Fig. 3a, b). Consistent with these results, the weight of tumors isolated from MIV-Luc-shIL13Rα2-injected cells was found to be reduced compared with those from control cells (Fig. 3c). Subsequent immunohistochemical analysis confirmed reduction of IL13Rα2 levels in tumors, whereas H&E staining did not reveal any major differences between shSCR and shIL13Rα2#2 primary tumors. Both exhibited similar morphology indicative of poorly differentiated carcinomas with cords and nests of moderately sized malignant cells and showed nuclei displaying focal nuclear pleomorphism (Fig. 3d). On the other hand, Ki67 staining revealed modest reduction in mitotic activity of IL13Rα2-depleted tumors consistent with the corresponding reduction in tumor weight (Fig. 3e).

**Fig. 4:**
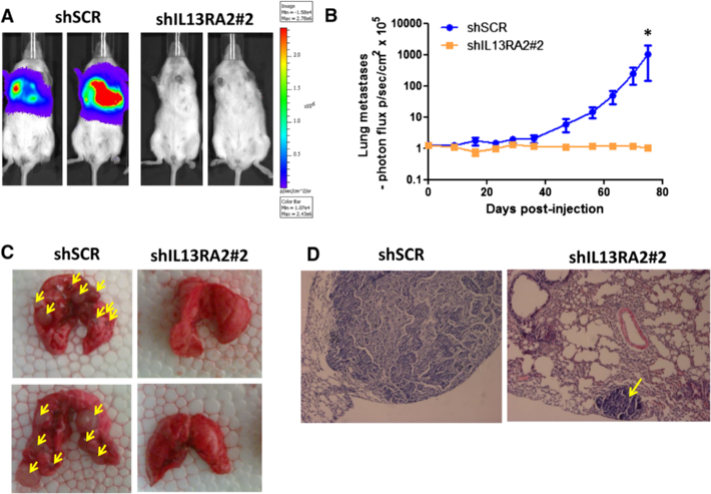
Targeting IL13Rα2 suppresses breast cancer metastasis to the lungs. **a** Representative endpoint (11 weeks) bioluminescence images of non-obese diabetic/severe combined immunodeficient mice injected intravenously with 5×10^5^ MIV-Luc-shSCR or MIV-Luc-shIL13Rα2#2 cells. **b** Quantification of photon flux (photons/sec per cm^2^) and comparison of metastatic colonization in the lungs of mice injected with MIV-Luc-shSCR or MIV-Luc-shIL13Rα2#2 cells (Mann-Whitney test, *P* value = 0.029, *n* = 4). *P < 0.05. **c** Representative images of lungs excised after euthanasia of mice injected with MIV-Luc-shSCR or MIV-Luc-shIL13Rα2#2 cells. *Yellow arrows* depict the presence of large macrometastatic nodules. **d** Representative images from hematoxylin-and-eosin staining of 10 μm-thick sections from paraffin-embedded lungs isolated from mice injected with MIV-Luc-shSCR or MIV-Luc-shIL13Rα2#2 cells. *Yellow arrow* indicates the presence of a micrometastatic lesion in a lung from MIV-Luc-IL13Rα2#2-injected mice (10× magnification). *IL13Rα2* interleukin-13 receptor alpha 2, *shIL13Rα2* small hairpin RNA against interleukin-13 receptor alpha 2, *shSCR* scrambled small hairpin RNA

Furthermore, to investigate whether overexpression of IL13Rα2 is involved in breast cancer metastasis, we performed in vivo metastasis assays by injecting MIV-Luc-shSCR or MIV-Luc-shIL13Rα2#2 cells via the lateral tail vein of NOD/SCID mice and quantified photon flux weekly by bioluminescence imaging. All mice were euthanized at 11 weeks post-injection, when control animals developed symptoms of cachexia, and lungs were removed to assess the presence of metastatic colonies. Remarkably, we found that whereas mice injected with the control MIV-Luc cells developed numerous large metastatic nodules in the lungs, none of the mice injected with the IL13Rα2-depleted MIV-Luc cells had any visible signs of macrometastases. This was clearly evident both by measuring the photon flux (Fig. 4a, b) and by gross examination of internal organs, including the lungs (Fig. 4c). Interestingly, histological analysis occasionally revealed the presence of micrometastatic lesions in the lungs of mice injected with the MIV-Luc-shIL13Rα2#2 cells (Fig. 4d). This evidence suggests that knockdown of IL13Rα2 either impairs extravasation of MIV cells in the lungs, or reduces survival in the circulation, or suppresses the colonization of secondary organs.

### Targeting IL13Rα2 induces TP63 expression and is associated with suppression of breast cancer cell migration

**Fig. 5:**
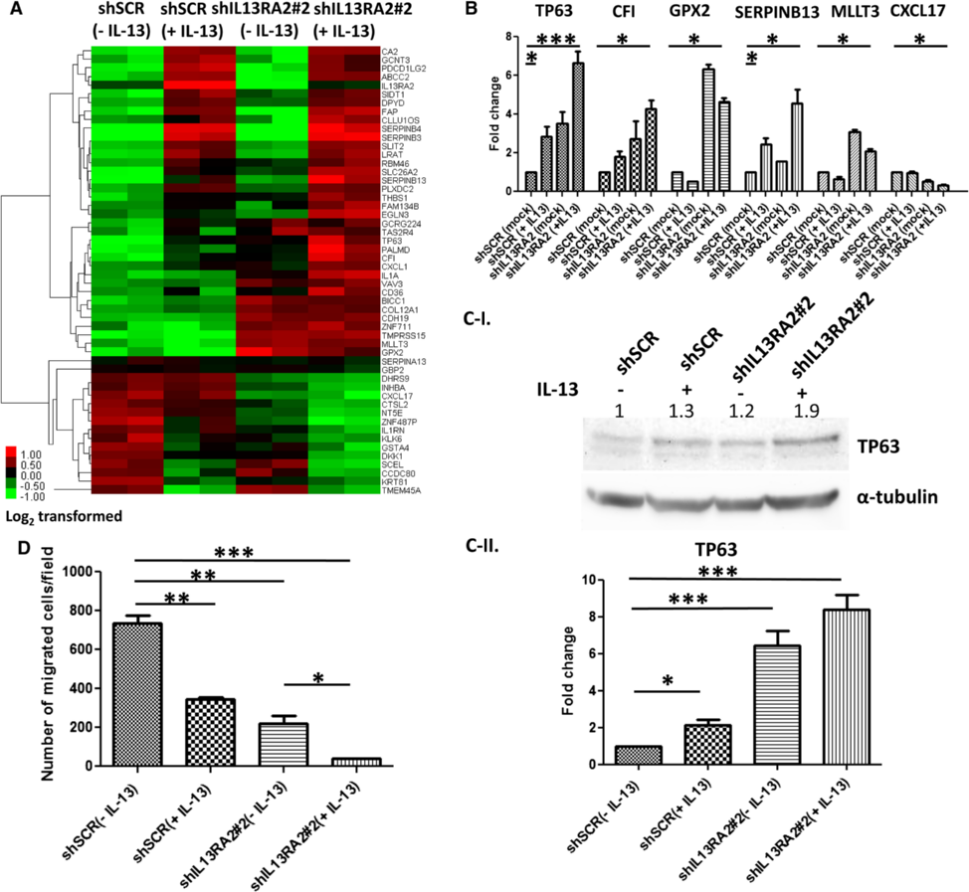
IL-13 signaling induces expression of the metastasis suppressor *TP63* and impairs cancer cell migration. **a** Heatmap showing the expression pattern, in all biological groups, of the 52 differentially expressed genes (35 upregulated and 17 downregulated) between MIV-Luc-shSCR (− IL-13) cells and MIV-Luc-shIL13Rα2#2 (+ IL-13, 20 ng/ml) cells. Values are expressed as log_2_-transformed. **b** Real-time PCR analysis for the validation of *TP63*, *CFI*, *GPX2*, *SERPINB13*, *MLLT3*, and *CXCL17* gene expression pattern. *P < 0.05, ***P < 0.001. **c**-**i** Western blotting analysis to assess the protein expression pattern of TP63 in the above-described conditions in MIV cells. **c**-**ii** Real-time PCR analysis was performed to measure *TP63* mRNA levels in SUM159-shSCR or SUM159-shIL13Rα2#2 cells in the presence or absence of 20 ng/ml IL-13 for 24 h. *P < 0.05, ***P < 0.001. **d** Transwell migration assays to quantify the migratory potential of MIV-Luc-shSCR and MIV-Luc-shIL13Rα2#2 cells in the presence or absence of 20 ng/ml IL-13 for 48 h. *P < 0.05, **P < 0.01, ***P < 0.001. *IL*-*13* interleukin-13, *PCR* polymerase chain reaction, *shIL13Rα2* small hairpin RNA against interleukin-13 receptor alpha 2, *shSCR* scrambled small hairpin RNA, *TP63* tumor protein p63

The dramatic suppression of lung metastasis formation upon IL13Rα2 knockdown led us to investigate the molecular mechanisms underlying this phenomenon. Because MIV cells harbor gain of the 5q31 locus resulting in an extra IL-13 gene copy [36], we hypothesized that IL-13 stimulates these cells in vivo in an autocrine fashion. First, we performed gene expression profiling of MIV cells to identify the differentially expressed genes in the presence or absence of IL13Rα2 or its ligand IL-13 or both. Initially, we aimed to identify genes which are transcriptionally regulated by IL-13 in breast cancer cells. The comparison of expression patterns between MIV-Luc-shSCR (− IL-13) and MIV-Luc-shSCR (+ IL-13) led to the identification of 28 differentially expressed genes (20 up- and 8 down-regulated) (Additional file 11: Table S1). To define the genes which are transcriptionally affected by high IL13Rα2 levels, we compared MIV-Luc-shSCR (− IL-13) and MIV-Luc-shIL13Rα2#2 (− IL-13) cells and revealed 36 differentially expressed genes (29 up- and 7 downregulated) (Additional file 12: Table S2). Because IL13Rα2 functions as a decoy receptor of IL-13 signaling, we hypothesized that a subset of critically important genes mediating the suppression of lung metastases are controlled by this signaling cascade. To identify such genes in our system, we performed unsupervised hierarchical clustering by comparing the differentially expressed genes between MIV-Luc-shSCR (− IL-13) and MIV-Luc-shIL13Rα2#2 (+ IL-13) (Fig. 5a). Within the 52 differentially expressed genes identified (36 up- and 16 downregulated), we selected six genes on the basis of exhibiting the following distinct expression pattern, consistent with the role of IL13Rα2 as a negative regulator of IL-13 signaling; for example, genes induced by this pathway should be upregulated in both MIV-Luc-shSCR (+ IL-13) and MIV-Luc-shIL13Rα2#2 (− IL-13) compared with untreated control cells and their expression levels should be additively or further enhanced in the MIV-Luc-shIL13Rα2#2 (+ IL-13) group. In addition, the list of genes that were differentially expressed on the basis of the pattern explained above was further refined, and *TP63*, *CFI*, *GPX2*, *SERPINB13*, *MLLT3*, and *CXCL17* genes were finally selected for validation by real-time PCR (Fig. 5b) on the basis of their previously reported roles in cancer progression. Interestingly, we found that the known metastasis suppressor *TP63* was among the top differentially expressed genes, suggesting that it may be a downstream target of IL-13 signaling with functional implications. We also examined TP63 protein levels by Western blotting and found that it is cooperatively induced by *IL13Rα2* knockdown and IL-13 in MIV cells (Fig. 5c-i). To further support these findings, stable depletion of *IL13Rα2* in the metastatic basal-like SUM159 cell line enhanced *TP63* levels in a similar manner (Fig. 5c-ii), whereas *IL13Rα2* overexpression in the non-metastatic MCF10A (MI) cells suppressed *TP63* mRNA and protein expression (Additional file 6: Figure S5c, S5d). Because decreased TP63 levels have been associated with enhanced invasion and migration as well as metastatic potential of cancer cells [37–39], we hypothesized that, upon IL13Rα2 knockdown, IL-13 signaling suppresses metastasis by impairing the ability of breast cancer cells to migrate. To address this question, we performed transwell migration assays which confirmed that IL13Rα2 depletion accompanied by IL-13 treatment could additively reduce the migration rate of metastatic MIV cells (Fig. 5d and Additional file 7: Figure S6). Importantly, the decrease in the ability of breast cancer cells to migrate was inversely proportional to TP63 expression levels (Fig. 5b, c), consistent with the role of TP63 as a suppressor of cell migration and metastasis. In contrast, knockdown of *IL13Rα2* had no effect on in vitro cell proliferation (Additional file 8: Figure S7a), anchorage-independent growth (Additional file 8: Figure S7b), or cell death due to loss of extracellular anchorage (Additional file 8: Figure S7c). Further supporting our in vitro findings, direct inoculation of MIV-Luc-shSCR or MIV-Luc-shIL13Rα2#2 cells in the right lung of NOD/SCID mice did not reveal any major differences in tissue colonization or survival between the two groups of animals (Additional file 9: Figure S8a, S8b). Collectively, this evidence suggests that targeting IL13Rα2 is likely to suppress metastasis, at least in part, by reducing cancer cell migration and inhibiting extravasation to the lungs.

### Activation of a STAT6-TP63 signaling axis by IL-13 is associated with increased survival of patients with breast cancer

**Fig. 6:**
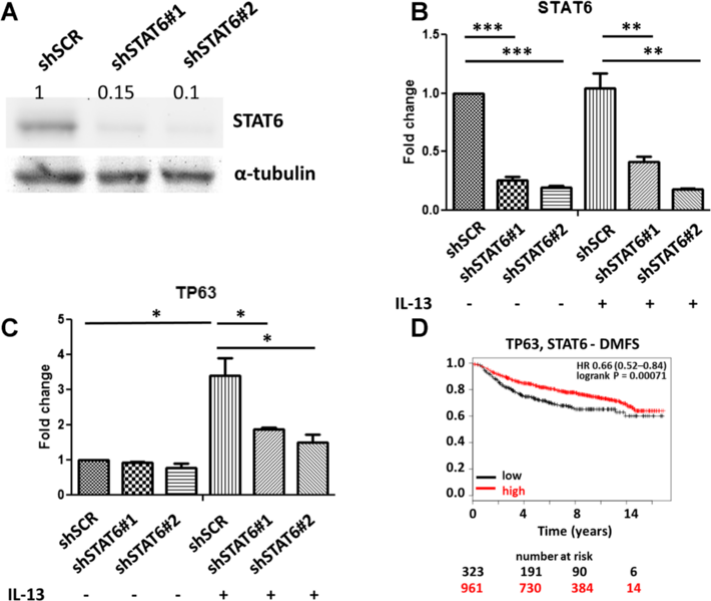
An IL-13-STAT6-TP63 signaling axis is associated with improved survival of patients with breast cancer. **a** MIV cells were stably transduced with shSCR or shSTAT6 lentiviral vectors expressing two different small hairpin RNA oligos (#1 and #2). Western blotting was performed in to measure STAT6 protein levels. The protein levels of α-tubulin were used as loading control. MIV-shSCR and MIV-shSTAT6 cells were treated with 20 ng/ml IL-13 for 24 h, and real-time polymerase chain reaction was performed to measure expression levels of either *STAT6* (**b**) (**P < 0.01, ***P < 0.001) or *TP63* (**c**) (*P < 0.05). **d** Kaplan-Meier analysis for assessment of DMFS in 1284 patients with breast cancer, based on combined expression of *STAT6* and *TP63*. Survival curves were generated by using the Kaplan-Meier Plotter online tool based on data stratified based on the median. Curves were compared by log-rank test. *DMFS* distant metastasis-free survival, *IL*-*13* interleukin-13, *shSCR* scrambled small hairpin RNA, *shSTAT6* small hairpin RNA against signal transducer and activator of transcription 6, *STAT6*, signal transducer and activator of transcription 6, *TP63* tumor protein p63

Because our data indicated that IL-13 signaling induces TP63 expression and that *IL13Rα2* depletion enhances IL-13-mediated STAT6 phosphorylation, we hypothesized that TP63 upregulation could be linked to STAT6 activation. To address whether STAT6 is implicated in TP63 expression, we first stably knocked down STAT6 in MIV cells and confirmed its decrease in both mRNA and protein levels (Fig. 6a, b). Importantly, we found that although IL-13 was able to induce *TP63* expression in MIV-Luc-shSCR cells, this effect was abolished upon STAT6 depletion (Fig. 6c). These data indicated that IL-13-mediated *TP63* transcription is directly or indirectly dependent on STAT6. Finally, based on this evidence, we hypothesized that if a metastasis suppressing IL-13-STAT6-TP63 signaling axis is activated in the absence of IL13Rα2 then *STAT6* and *TP63* expression levels may coordinately predict survival of patients with breast cancer. Indeed, Kaplan-Meier plotting analysis revealed that patients with higher average expression of STAT6 and TP63 have significantly longer DMFS and relapse-free survival (RFS) compared with patients with low expression levels of these genes (Fig. 6d and Additional file 10: Figure S9).

## Discussion

Metastasis is the major cause for the vast majority of deaths of patients with breast cancer. BLBCs are among the most aggressive subtypes and often share similar features with triple-negative tumors. The common ineffectiveness of most available targeted therapies to treat metastatic basal-like tumors highlights the urgent need for the identification of new therapeutic targets. To identify novel genes implicated in promoting breast cancer metastasis, we took advantage of a well-established cell line system for BLBC progression. Gene expression profiling of the model cell lines along with mining of pre-existing tumor transcriptome data revealed that IL13Rα2 overexpression is significantly associated with basal compared with luminal tumor subtype as well as bad prognosis and is also overexpressed in a subset of highly metastatic basal-B cancer cell lines. Kaplan-Meier survival analysis further indicated that increased IL13Rα2 levels are associated with shorter metastasis-free survival of patients with high-grade breast tumors. Importantly, depletion of IL13Rα2 resulted in a modest but consistent delay in primary tumor growth and dramatically suppressed lung metastasis formation in vivo. Consistent with our findings, there is a growing list of cancers in which overexpression of IL13Rα2 has been reported to promote tumor progression, including glioblastoma, pancreatic, ovarian, and colon cancers, suggesting a critical role for this alteration in a multitude of cancers [18, 20–22].

To decipher the molecular basis for how targeting IL13Rα2 suppressed breast cancer metastasis, we examined the phosphorylation status of STAT proteins and found enhanced STAT6 phosphorylation as the major downstream signaling mediator. Subsequent gene expression profiling of metastatic breast cancer cells proficient or deficient in IL13Rα2, and treated with or without IL-13, identified a subset of genes regulated by the IL-13 signaling pathway. Interestingly, the known metastasis suppressor gene *TP63* was among the genes found to be significantly upregulated by IL-13 in the context of *IL13Rα2* depletion. Previous studies have shown that, whereas the truncated ΔNP63 isoform can promote tumor growth [40, 41], loss of the full-length *TP63* expression enhances invasion and migration of cancer cells as well as their ability to metastasize to distal organs [37–39]. Moreover, knockdown of *STAT6* abolished the ability of IL-13 to induce *TP63* expression, suggesting that it is involved, directly or indirectly, in its transcriptional activation. Consistent with the role of TP63 as a suppressor of breast cancer cell migration, the combination of *IL13Rα2* depletion concomitant with IL-13 treatment potently suppressed the migratory potential of metastatic MIV cells. In contrast, the effect of *IL13Rα2* in regulating metastasis appears to be independent of cell proliferation, anchorage-independent growth, anoikis, or in vivo colonization to the lungs. Therefore, the fact that depletion of STAT6 impaired *TP63* upregulation suggests that a STAT6-TP63 axis may be involved, at least in part, in IL-13-mediated suppression of cancer cell migration, extravasation, and eventually metastasis. However, we cannot rule out the possible involvement of other genes identified in our expression profiling analysis as candidates for suppressing metastasis via the IL-13 pathway. It is noteworthy that we identified not only genes previously reported to be regulated by IL-13, such as *CD36* [42, 43], *SERPINB3*, and *SERPINB4* [44], but also a number of other genes that have not been previously connected to this pathway. It is possible that some of these, such as the orphan chemokine CXCL17, recently shown to promote angiogenesis and cancer progression [45], could also be involved in regulating metastatic dissemination via the IL-13 pathway. Additionally, among the known IL-13 regulated genes, *SERPINB13* is another legitimate candidate as its low expression has been associated with poor clinical outcome of head and neck cancers [46]. Additional functional studies will be necessary to elucidate the potential involvement of such candidate effector genes in metastatic progression downstream of IL-13.

Overall, our studies resulted in the unbiased discovery that IL13Rα2 overexpression is a major contributor to metastatic progression in a subset of BLBCs. We also provide evidence, for the first time, for the existence of an anti-migratory IL-13-STAT6-TP63 signaling axis that may exert metastasis-suppressing effects on breast cancer cells. This evidence is further supported by the Kaplan-Meier survival plotting analysis which indicated that high levels of *STAT6* and *TP63* expression were associated with longer DMFS of patients with breast cancer.

## Conclusions

Cell membrane receptors that facilitate breast cancer metastasis represent an attractive group of accessible molecular targets that could be exploited as the basis for personalized therapy. Therefore, the discovery of IL13Rα2 as an important mediator of metastatic disease in a subset of aggressive BLBCs should be further evaluated both as a biomarker and as a potentially important therapeutic target for preventing cancer metastasis.

## Additional files

Additional file 1: **Supplementary materials and methods.** Detailed description of materials and methods used for experiments that are described in the supplementary figures.

Additional file 2: Figure S1. Gene expression profiling of the MII, MIII, and MIV cell lines. Heatmap showing the gene expression pattern between MII, MII, and MIV breast cancer cells as we describe elsewhere [32]. Adjacent heatmap focuses on cluster 9, which includes 29 genes with higher expression levels in MIV compared with MIII and MII cells. Heatmap colors indicate the z-score for the expression of each gene (*red*, highest expression; *blue*, lowest expression).

Additional file 3: Figure S2. Kaplan-Meier survival plot analysis of candidate metastasis-promoting genes. Kaplan-Meier survival plots were generated by using the Kaplan-Meier Plotter online tool [24] based on data stratified at either the lower quartile or the median expression of each gene, as indicated. (**a**) Distant metastasis-free survival (DMFS) analysis of patients for all breast cancers was calculated on the basis of tumor IL13Rα2 expression for each of the following genes: *SMTN*, *AGTPBP1*, *IGF2BP2*, *VEGFA*, *HMGCS1*, *TRIB3*, *INSIG1*, *TMEM97*, *INHBA*, and *IL13RA2*. Curves were compared by log-rank test. (**b**) DMFS analysis of patients for different tumor grades (1, 2, or 3) was calculated on the basis of tumor IL13RA2 expression for each of the following genes: *SMTN*, *AGTPBP1*, *IGF2BP2*, *VEGFA*, *HMGCS1*, *TRIB3*, *INSIG1*, *TMEM97*, and *INHBA*. Curves were compared by log-rank test. *IL13Rα2* interleukin-13 receptor alpha 2.

Additional file 4: Figure S3. Meta-analysis of breast cancer cell line gene expression. Meta-analysis of previously published microarray data [23, 29] from 27 luminal, 12 basal-A, and 11 basal-B breast cancer cell lines to assess the expression levels of *IL13Rα2*. Fold change in *IL13Rα2* levels was calculated compared with the expression of the non-tumorigenic MCF10A cell line. *IL13Rα2* interleukin-13 receptor alpha 2.

Additional file 5: Figure S4. Evaluation of IL13Rα2 levels in breast cancer cell lines. Western blotting analysis (**a**) and real-time PCR (**b**) were performed to measure IL13Rα2 protein and mRNA levels, respectively, in MI, MII, MIII, and MIV cells. (**c**) Western blotting analysis was performed to measure basal IL13Rα2 protein in SUM159, parental MDA-MB-231, and MDA-MB-231-LM2 cells. (**d**) Real-time PCR analysis to quantify IL13Rα2 mRNA levels in SKBR3, MCF7, MDA-MB-361, MDA-MB-231, SUM159, and MDA-MB-231-LM2 cells. *P < 0.05, **P < 0.01, ***P < 0.001. *IL13Rα2* interleukin-13 receptor alpha 2, *ND* not detected, *PCR* polymerase chain reaction.

Additional file 6: Figure S5. IL13Rα2 overexpression suppresses IL-13-mediated STAT6 phosphorylation and TP63 expression. (**a**) MCF10A (MI) cells were transfected either empty pCMV6 vector or pCMV6- IL13Rα2-Myc-Flag construct and 48 h post-transfection were either mock-treated or treated with 20 ng/ml IL-13 for additional 24 h. Real-time PCR analysis was performed to measure IL13Rα2 mRNA levels. (**b**) MCF10A (MI) cells were transfected either empty pCMV6 vector or pCMV6- IL13Rα2-Myc-Flag construct and 48 h post-transfection were either mock-treated or treated with 1 ng/ml IL-13 for 30 min. Western blotting analysis was performed in whole cell lysates to measure P-STAT6 (Y641) levels. Total STAT6 and GAPDH protein levels were also measured as loading controls. (**c**) MCF10A (MI) cells were transfected either empty pCMV6 vector or pCMV6-IL13Rα2-Myc-Flag construct and 48 h post-transfection were either mock-treated or treated with 20 ng/ml IL-13 for an additional 24 h. Real-time PCR analysis (**c**) or Western blotting analysis (**d**) was performed to measure TP63 mRNA or protein levels, respectively. Total STAT6 and GAPDH protein levels were measured as loading controls where indicated. *IL*-*13* interleukin 13, *IL13Rα2* interleukin-13 receptor alpha 2, *PCR* polymerase chain reaction, *STAT6* signal transducer and activator of transcription 6, *TP63* tumor protein p63.

Additional file 7: Figure S6. IL13Rα2 knockdown does not affect in vitro cell proliferation, anchorage-independent growth, or anoikis. (**a**) MIV-Luc-shSCR or MIV-Luc-shIL13RA2 cells (4×10^3^) were seeded in 96-well culture plates in 100 μl complete medium, incubated at 37 °C in a humidified incubator with 5 % CO_2_, and allowed to grow up to 96 h. The number of viable cells at 24, 48, 72, or 96 h was determined by using an MTS assay and absorbance at 490 nm. (**b**) Soft agar colony formation assay was performed by seeding 5×10^3^ MIV-Luc-shSCR or MIV-Luc-shIL13RA2 cells in 0.35 % low melting agarose-medium solution on top of a 0.5 % base agar layer. Plates were incubated in a humidified CO_2_ incubator for 14 days. Colonies formed were stained by using 0.5 % crystal violet in 20 % methanol solution and counted. (**c**) MIV-Luc-shSCR or MIV-Luc-shIL13RA2 cells were seeded on a 96-well ultra-low attachment plate at low density (4×10^3^ or 8×10^3^ cells) in 100 μl complete medium. Cells were incubated at 37 °C in a humidified incubator with 5 % CO_2_ and allowed to grow for 48 or 96 h. The number of viable cells at 24, 48, 72, or 96 h was determined by using an MTS assay and absorbance at 490 nm. All experiments were performed in triplicates, and statistical significance was assessed by using Student’s *t* test. *IL13Rα2* interleukin-13 receptor alpha 2, *STAT6* signal transducer and activator of transcription 6, *TP63* tumor protein p63.

Additional file 8: Figure S7 IL13Rα2 silencing does not affect breast cancer cell colonization in the lungs. (**a**) Comparison of the number of macroscopic nodules in the right lung of non-obese diabetic/severe combined immunodeficient mice injected with 5×10^5^ MIV-Luc-shSCR or MIV-Luc-shIL13Rα2#2. Cells were inoculated directly into the right lung of mice by injection into the upper margin of the sixth intercostal rib on the right anterior axillary line, as previously described [47]. Animals (*n* = 4) were monitored daily over a period of up to 52 days, and each mouse was euthanized when it developed notable cachexia symptoms. When mice were sacrificed, the lungs were excised and the number of macroscopic nodules in the right lung was counted and compared between the two groups. (**b**) Survival curves for the two animal groups were generated on the basis of the days that mice were euthanized. Statistical significance was assessed by using Student’s *t* test. *IL13Rα2* interleukin-13 receptor alpha 2, *NS* not significant, *shIL13Rα2* small hairpin RNA against interleukin-13 receptor alpha 2, *shSCR* scrambled small hairpin RNA.

Additional file 9: Figure S8. IL13Rα2 depletion and IL-13 treatment additively suppress breast cancer cell migration. Representative images from transwell migration assays. Cells proficient or deficient in IL13Rα2, treated with 20 ng/ml IL-13 for 48 h or not, that were localized on the bottom membrane surface were stained with trypan blue (0.4 %) in order to quantify their migratory potential. *IL*-*13* interleukin-13, *IL13Rα2* interleukin-13 receptor alpha 2.

Additional file 10: Figure S9. Kaplan-Meier survival plot analysis for STAT6 and TP63. (**a**) Kaplan-Meier relapse-free survival (RFS) plots for patients with breast cancer were generated by using the Kaplan-Meier Plotter online tool [24] based on data stratified based on the median tumor expression of STAT6 and TP63 individually or based on their median expression combined. (**b**) Distant metastasis-free survival (DMFS) analysis of patients with breast cancer, based on the median tumor expression of STAT6 and TP63 individually. All curves were compared by log-rank test. *STAT6* signal transducer and activator of transcription 6, *TP63* tumor protein p63.

Additional file 11: Table S1. List of differentially expressed genes between MIV-shSCR (−IL13) VS MIV-shSCR (+IL13) cells. *shSCR*scrambled small hairpin RNA.

Additional file 12: Table S2. List of differentially expressed genes between MIV-shSCR (−IL13) VS MIV-shIL13RA2 (−IL13) cells. *shIL13Rα2* small hairpin RNA against interleukin-13 receptor alpha 2, *shSCR* scrambled small hairpin RNA.

## Abbreviations

BLBC: Basal-like breast cancer
CD36: Cluster of differentiation 36
CFI: Complement factor I
CXCL17: Chemokine (C-X-C motif) ligand 17
DMEM: Dulbecco’s modified Eagle’s medium
DMFS: Distant metastasis-free survival
ER: Estrogen receptor
FBS: Fetal bovine serum
GPX2: Glutathione peroxidase 2
H&E: Hematoxylin and eosin
Her2: Human epidermal growth factor 2
IL-4: Interleukin 4
IL4Rα: Interleukin 4 receptor alpha
IL-13: Interleukin-13
IL13Rα2: Interleukin-13 receptor alpha 2
JAK: Janus kinase 2
Luc: Luciferase
MLLT3: Mixed-lineage leukemia (Trithorax homologue) 3
MSKCC: Memorial Sloan Kettering Cancer Center
NOD/SCID: Non-obese diabetic/severe combined immunodeficient
PCR: Polymerase chain reaction
PR: Progesterone receptor
SERPINB3: Serpin B3
SERPINB4: Serpin B4
SERPINB13: Serpin B13
shIL13Rα2: Small hairpin RNA against interleukin-13 receptor alpha 2
shRNA: Small hairpin RNA
shSCR: scrambled small hairpin RNA
shSTAT6: small hairpin RNA against signal transducer and activator of transcription 6
STAT6: Signal transducer and activator of transcription 6
TP63: Tumor protein p63
