# Supplementary material for: Targeting IL13Ralpha2 activates STAT6-TP63 pathway to suppress breast cancer lung metastasis
Source: Breast Cancer Res. 2015 Jul 25;17(1):98. doi: 10.1186/s13058-015-0607-y (PMC4531803; doi:10.1186/s13058-015-0607-y)
Supplement: Supplementary file 1 — Supplementary materials and methods. Detailed description of materials and methods used for experiments that are described in the supplementary figures. [file 13058_2015_607_MOESM1_ESM.pdf]

Supplementary materials and methods:

In vitro cell proliferation assay:

MIV-Luc-shSCR or MIV-Luc-shIL13RA2 cells ($4x10^3$) were seeded on a 96-well tissue culture-treated plate (Corning 3904) in 100ul complete medium containing 2µg/ml puromycin to maintain knockdown. Cells were incubated at 37°C in a humidified incubator with 5% CO_2 and allowed to grow up to 96h. MTS/PMS solution (20µl) (Promega G5421) was then added to each well and the plate was incubated at 37°C for 1 hour. Absorbance at 490nm was then measured to quantify the amount of viable cells in each well. These experiments were performed in triplicates and statistical significance was assessed using student's t-test.

Colony formation soft agar assay:

Soft agar plates were prepared by mixing 1% low melting agarose (DNA grade) with warm 2x DMEM F/12 complete medium in order to give 0.5% agarose-medium. The mixture was poured in 60mm culture-treated plates and allowed to solidify in order to form a 0.5% base agar. MIV-Luc-shSCR or MIV-Luc-shIL13RA2 cells were trypsinized, counted and $5x10^3$ cells were mixed with similarly prepared 0.35% agarose-medium mix (top agar). Plates were incubated in a humidified CO_2 incubator for 14 days. Colonies formed were stained using 0.5% crystal violet in 20% methanol solution and counted. These experiments were performed in triplicates and statistical significance was assessed using student's t-test.

Anoikis-induction assay:

MIV-Luc-shSCR or MIV-Luc-shIL13RA2 cells were seeded on a 96-well ultra-low attachment plate (Corning 3474) at low density ($4x10^3$ or $8x10^3$ cells) in 100µl complete medium containing 2µg/ml puromycin to maintain gene knockdown. Cells were incubated at 37°C in a humidified incubator with 5% CO_2 and allowed to grow for 48h or 96h. MTS/PMS solution (20µl) (Promega)

was then added to each well and the plate was incubated at 37°C for 1 hour. Absorbance at 490nm was then measured to quantify the amount of viable cells in each well. These experiments were performed in triplicates and statistical significance was assessed using student's t-test.

Breast cancer cell lung implantations: Inoculation of breast cancer cells directly in the lungs was performed as previously described by Liu X. et al., *J Thorac Dis. 2012* [1]. Briefly, six week-old female NOD.CB17-*Prkdc*^{scid}/J mice (The Jackson Laboratory) were anesthetized by intraperitoneal injection of Avertin solution (250mg/Kg). MIV-Luc-shSCR or MIV-Luc-shIL13RA2#2 cells ($5X10^5$) were trypsinized, resuspended in 100µl serum-free DMEM F/12 medium and injected into the upper margin of the sixth intercostal rib on the right anterior axillary line to a depth of about 5-6 mm using a 30G needle. Animals (n=4) were then monitored daily over a period of up to 52 days and each mouse was euthanized when developed notable cachexia symptoms. When mice were sacrificed, the lungs were excised and the number of macroscopic nodules in the right lung was counted and compared between the two groups. Survival curves were also generated for mice injected either with MIV-Luc-shSCR or MIV-Luc-shIL13RA2#2 cells.

List of real-time PCR primers:

**VEGF**	**SMTN**
F: AGGCCAGCACATAGGAGAGA	F: CGAGTGAACAAAGCACCAGA
R: TTTCTTGCGCTTTCGTTTTT	R: ATGAGCTTCCGCTCTTCAAA
**IL13RA2**	**SERPINA3**
F: TCTTGGAAACCTGGCATAGG	F: GGCCCCTGATAAGAATGTCA
R: TCTGATGCCTCCAAATAGGG	R: AGCTCATCGCTGGACTGATT
**IGF2BP2**	**TMEM97**
F: TGAACATGAAACAGGGACCA	F: TTCTGTTTTGCGAGCTTGTG
R: TATCTCAGCACTGGCACAGG	R: GAAACCACTGGCTTTGGAGA

**INHBA**	**HMGCS1**
F: TTTCTGTTGGCAAGTTGCTG	F: GGGACACATATGCAACATGC
R: CGGGTCTCTTCTTCAAGTGC	R: CACTGGGCATGGATCTTTTT
**AGTPBP1**	**TRIB3**
F: CCCCACTGCTCAGAGCTTAC	F: TCCAGAAACGAGCTCGAAGT
R: TCACTGCAGCCAAGTATTGC	R: TGCACGATCTGGAGCAGTAG
**STAT6**	**TP63**
F: CAACCACTTCCTACCCCAGA	F: CTCCCCACCTCTGAACAAAA
R: ATGCTCATGGAGGAATCAGG	R: GCTGCTGAGGGTTGATAAGC
**CFI**	**GPX2**
F: TGGATGCCAACAATGTGACT	F: CCTTCACCCTTGTCCAAAAA
R: TGGGAACTCTGGTTTTCCAC	R: AAATGATGAGCTTGGGATCG
**SERPINB13**	**MLLT3**
F: TTGAGGTGGAGGACGGTTAC	F: GCTGGTTTCATTTTGCCAAT
R: TTGTGCTCACTGAAGGCATC	R: TAGCTTTTCACAGCGGAGGT
**CXCL17**	
F: TCATTTCAAGGGCAATGTGA	
R: TGCTTGTTTGGCTTTCTGTG	

shRNA oligos

**IL13RA2 oligoF1**
CCGG GCTTTCGTTTGCTTGGCTATCCTCGAGGATAGCCAAGCAAACGAAAGCTTTTTG
**IL13RA2 oligoR1**
AATTCAAAAA GCTTTCGTTTGCTTGGCTATCCTCGAGGATAGCCAAGCAAACGAAAGC
**IL13RA2 oligoF2**
CCGGGCTATCGGATGCTTATATACCCTCGAGGGTATATAAGCATCCGATAGCTTTTTG
**IL13RA2 oligoR2**
AATTCAAAAA GCTATCGGATGCTTATATACCCTCGAGGGTATATAAGCATCCGATAGC

**STAT6 oligo F1:**
CCGGGGTGCCTTCTTATGACCTTGGCTCGAGCCAAGGTCATAAGAAGGCACCTTTTTG
**STAT6 oligo R1:**
AATTCAAAAAGGTGCCTTCTTATGACCTTGGCTCGAGCCAAGGTCATAAGAAGGCACC
**STAT6 oligo F2:**
CCGGGGGCACTTGGATTGGTGAAGACTCGAGTCTTCACCAATCCAAGTGCCCTTTTTG
**STAT6 oligo R2:**
AATTCAAAAAGGGCACTTGGATTGGTGAAGACTCGAGTCTTCACCAATCCAAGTGCCC

References:

1. Liu X, Liu J, Guan Y, Li H, Huang L, Tang H, He J: Establishment of an orthotopic lung cancer model in nude mice and its evaluation by spiral CT. *J Thorac Dis* 2012, 4:141-145.
