# Supplementary figures and images for: Targeting IL13Ralpha2 activates STAT6-TP63 pathway to suppress breast cancer lung metastasis
Source: Breast Cancer Res. 2015 Jul 25;17(1):98. doi: 10.1186/s13058-015-0607-y (PMC4531803; doi:10.1186/s13058-015-0607-y)

# Metastatic potential

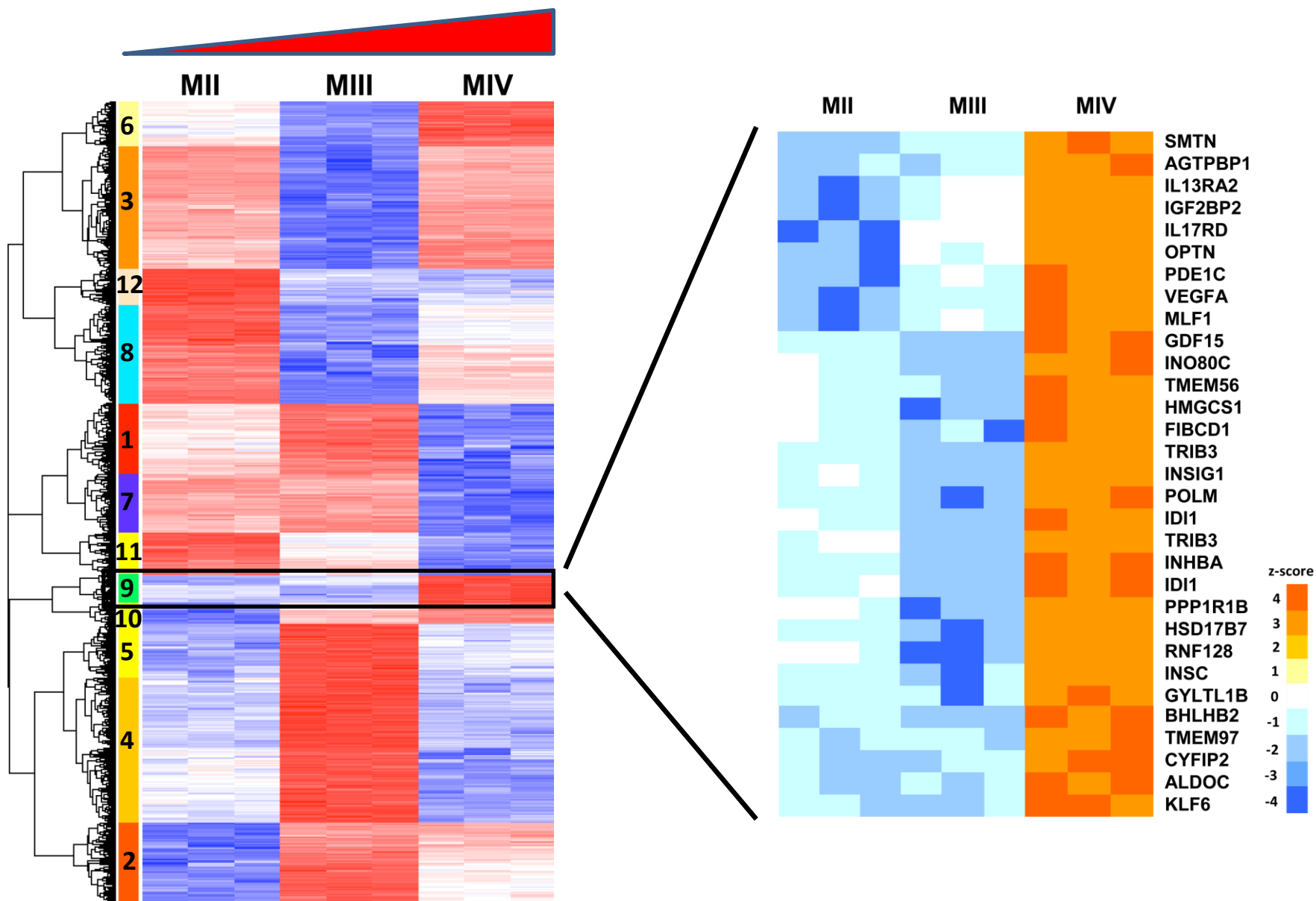

Supplement: Supplementary file 2 — Gene expression profiling of the MII, MIII, and MIV cell lines. Heatmap showing the gene expression pattern between MII, MII, and MIV breast cancer cells as we describe elsewhere [32]. Adjacent heatmap focuses on cluster 9, which includes 29 genes with higher expression levels in MIV compared with MIII and MII cells. Heatmap colors indicate the z-score for the expression of each gene (red, highest expression; blue, lowest expression). [file 13058_2015_607_MOESM2_ESM.pdf]

## Luminal

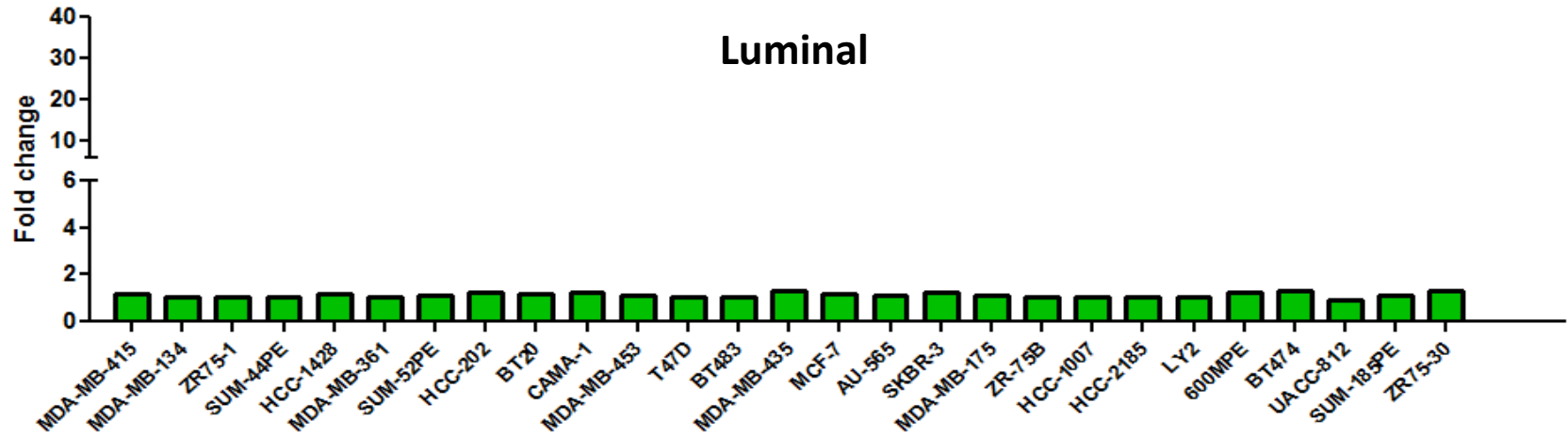

## Basal A

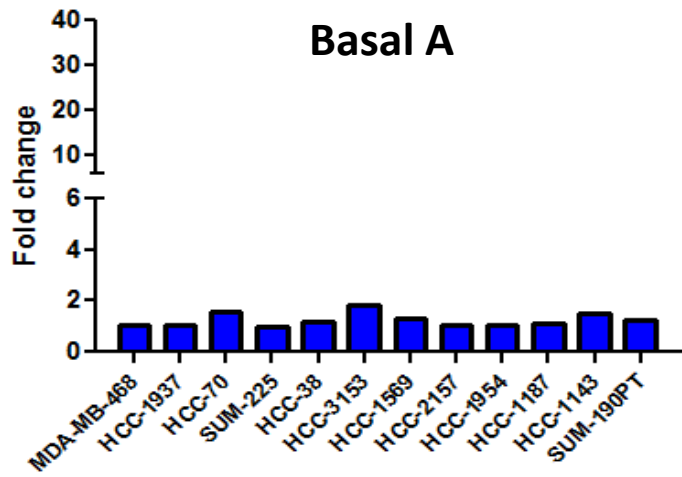

## Basal B

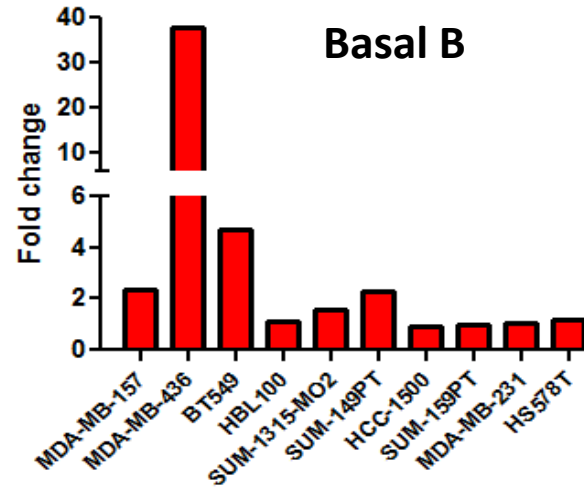

## Basal B

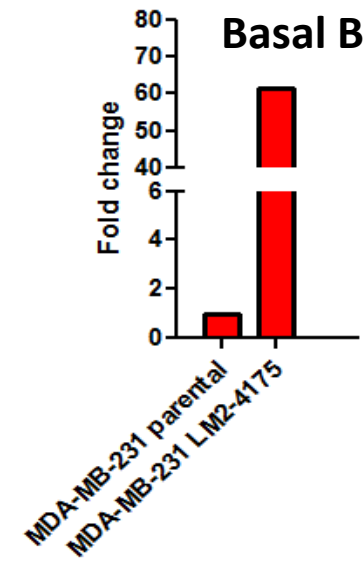

Supplement: Supplementary file 4 — Meta-analysis of breast cancer cell line gene expression. Meta-analysis of previously published microarray data [23, 29] from 27 luminal, 12 basal-A, and 11 basal-B breast cancer cell lines to assess the expression levels of IL13Rα2. Fold change in IL13Rα2 levels was calculated compared with the expression of the non-tumorigenic MCF10A cell line. IL13Rα2 interleukin-13 receptor alpha 2. [file 13058_2015_607_MOESM4_ESM.pdf]

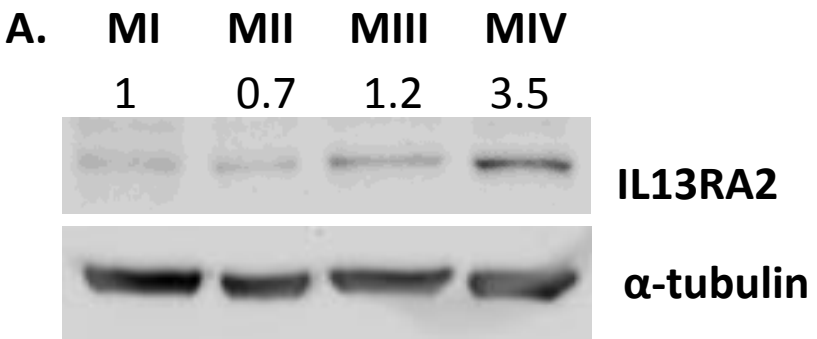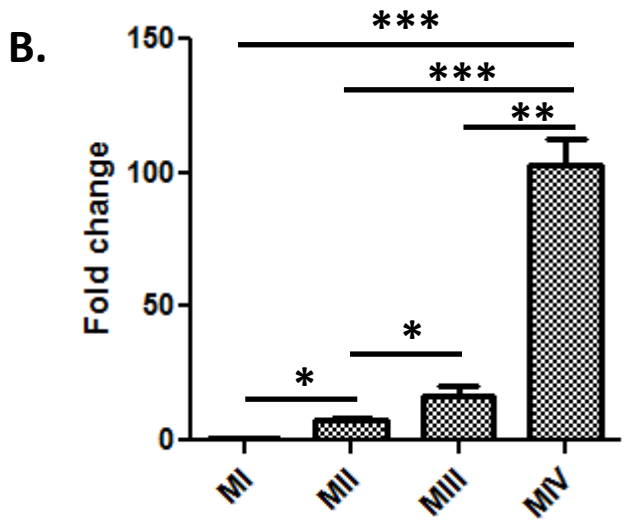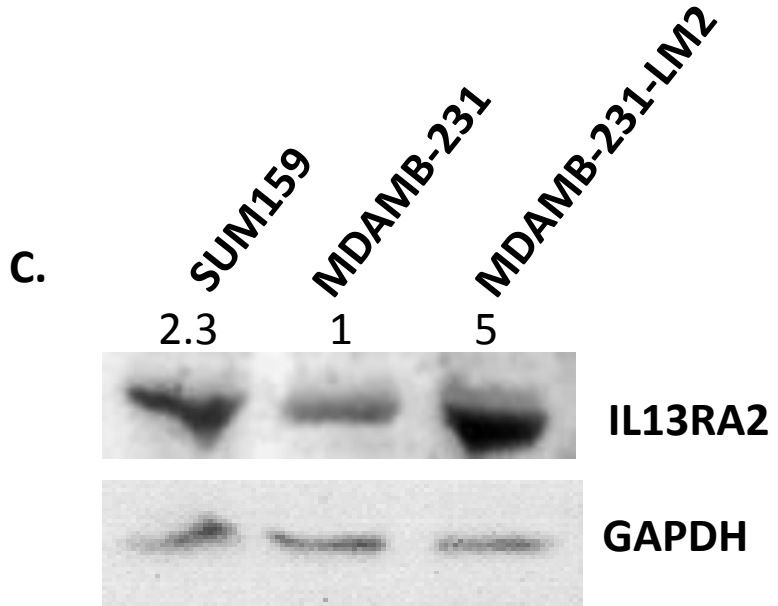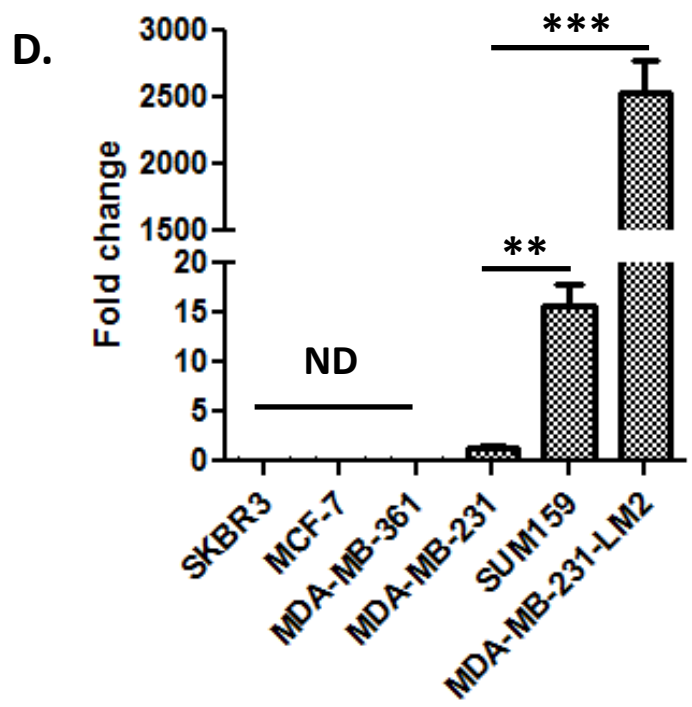

Supplement: Supplementary file 5 — Evaluation of IL13Rα2 levels in breast cancer cell lines. Western blotting analysis (a) and real-time PCR (b) were performed to measure IL13Rα2 protein and mRNA levels, respectively, in MI, MII, MIII, and MIV cells. (c) Western blotting analysis was performed to measure basal IL13Rα2 protein in SUM159, parental MDA-MB-231, and MDA-MB-231-LM2 cells. (d) Real-time PCR analysis to quantify IL13Rα2 mRNA levels in SKBR3, MCF7, MDA-MB-361, MDA-MB-231, SUM159, and MDA-MB-231-LM2 cells. *P < 0.05, **P < 0.01, ***P < 0.001. IL13Rα2 interleukin-13 receptor alpha 2, ND not detected, PCR polymerase chain reaction. [file 13058_2015_607_MOESM5_ESM.pdf]

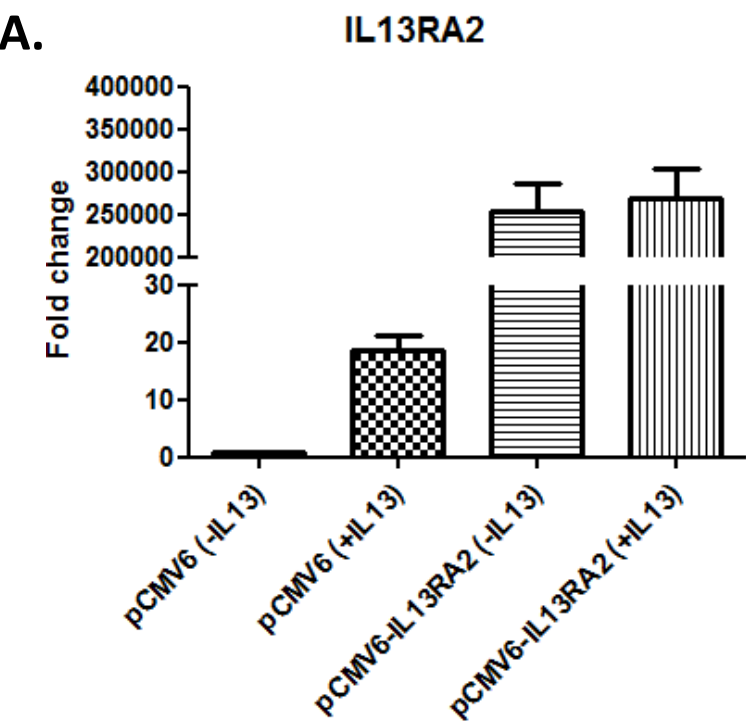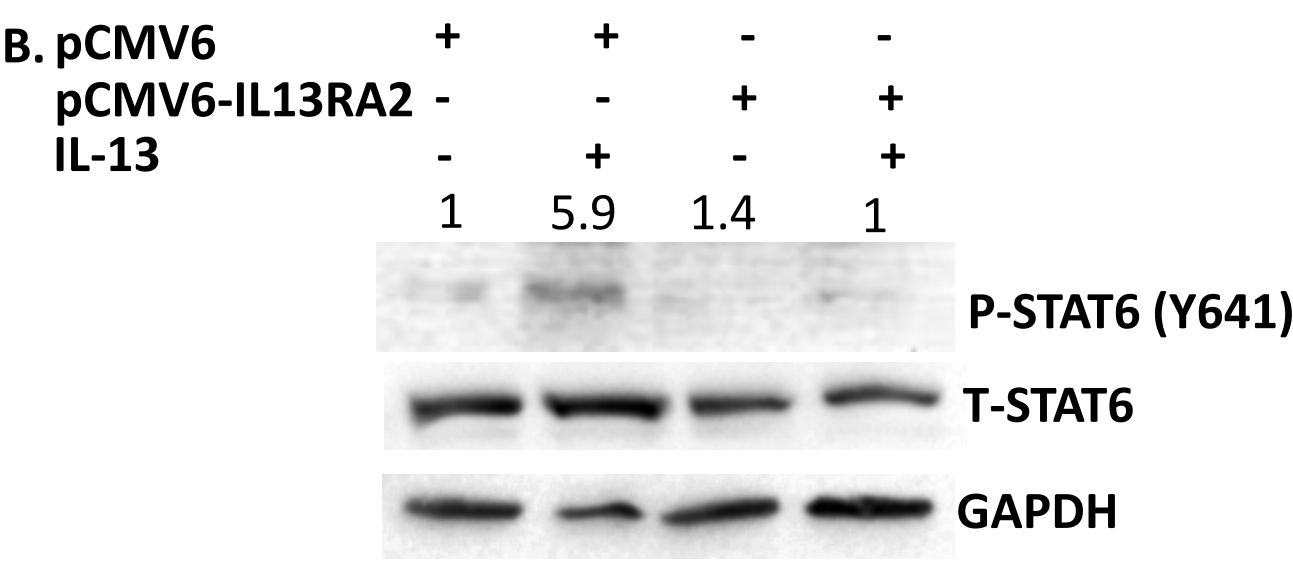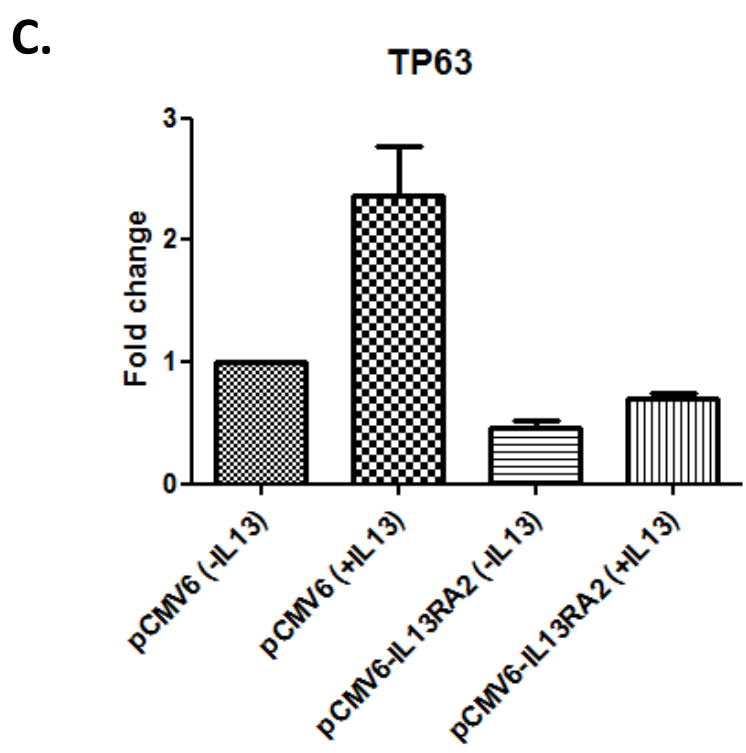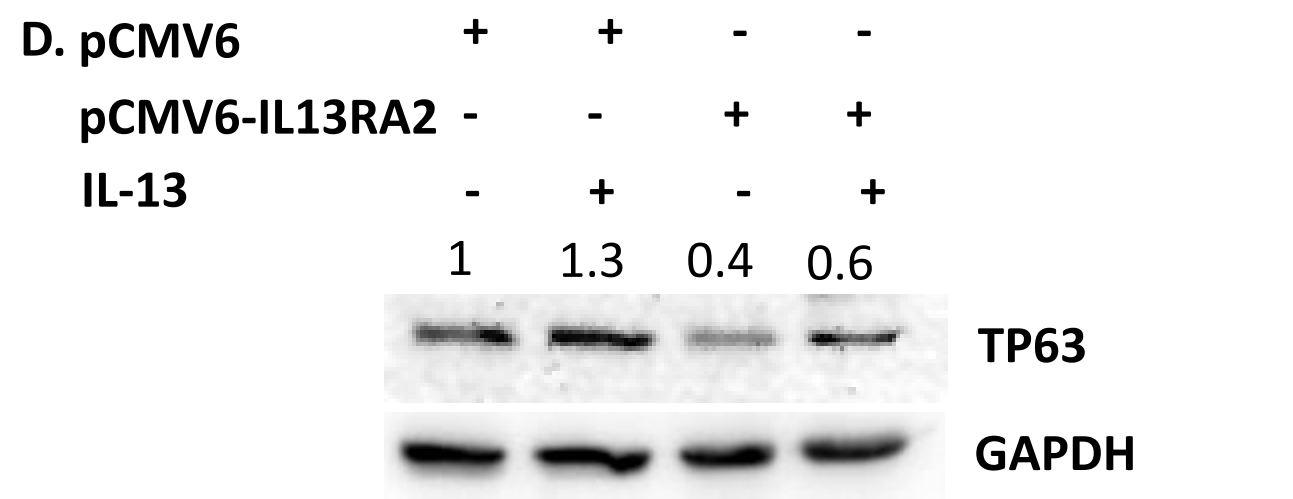

Supplement: Supplementary file 6 — IL13Rα2 overexpression suppresses IL-13-mediated STAT6 phosphorylation and TP63 expression. (a) MCF10A (MI) cells were transfected either empty pCMV6 vector or pCMV6- IL13Rα2-Myc-Flag construct and 48 h post-transfection were either mock-treated or treated with 20 ng/ml IL-13 for additional 24 h. Real-time PCR analysis was performed to measure IL13Rα2 mRNA levels. (b) MCF10A (MI) cells were transfected either empty pCMV6 vector or pCMV6- IL13Rα2-Myc-Flag construct and 48 h post-transfection were either mock-treated or treated with 1 ng/ml IL-13 for 30 min. Western blotting analysis was performed in whole cell lysates to measure P-STAT6 (Y641) levels. Total STAT6 and GAPDH protein levels were also measured as loading controls. (c) MCF10A (MI) cells were transfected either empty pCMV6 vector or pCMV6-IL13Rα2-Myc-Flag construct and 48 h post-transfection were either mock-treated or treated with 20 ng/ml IL-13 for an additional 24 h. Real-time PCR analysis (c) or Western blotting analysis (d) was performed to measure TP63 mRNA or protein levels, respectively. Total STAT6 and GAPDH protein levels were measured as loading controls where indicated. IL-13 interleukin 13, IL13Rα2 interleukin-13 receptor alpha 2, PCR polymerase chain reaction, STAT6 signal transducer and activator of transcription 6, TP63 tumor protein p63. [file 13058_2015_607_MOESM6_ESM.pdf]

**shSCR**

**shIL13RA2**

**SF**

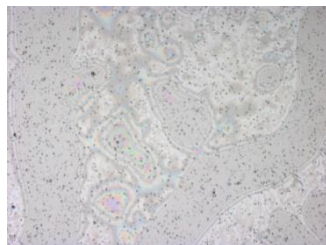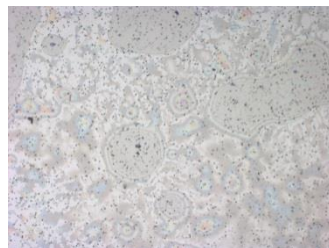

**10%  
FBS**

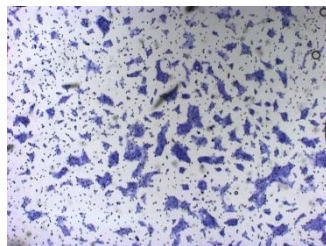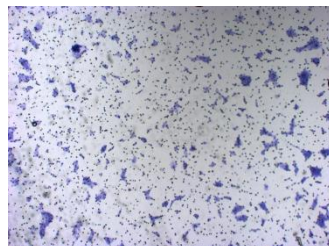

**- IL13**

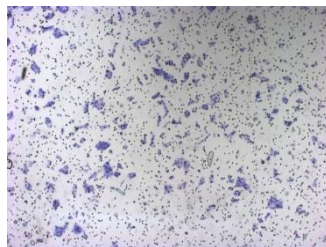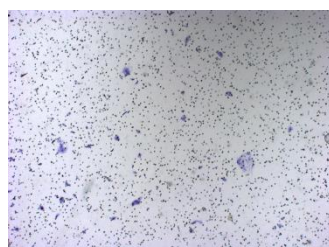

**+ IL13**

Supplement: Supplementary file 7 — IL13Rα2 knockdown does not affect in vitro cell proliferation, anchorage-independent growth, or anoikis. (a) MIV-Luc-shSCR or MIV-Luc-shIL13RA2 cells (4×10^3^) were seeded in 96-well culture plates in 100 μl complete medium, incubated at 37 °C in a humidified incubator with 5 % CO2, and allowed to grow up to 96 h. The number of viable cells at 24, 48, 72, or 96 h was determined by using an MTS assay and absorbance at 490 nm. (b) Soft agar colony formation assay was performed by seeding 5×10^3^ MIV-Luc-shSCR or MIV-Luc-shIL13RA2 cells in 0.35 % low melting agarose-medium solution on top of a 0.5 % base agar layer. Plates were incubated in a humidified CO2 incubator for 14 days. Colonies formed were stained by using 0.5 % crystal violet in 20 % methanol solution and counted. (c) MIV-Luc-shSCR or MIV-Luc-shIL13RA2 cells were seeded on a 96-well ultra-low attachment plate at low density (4×10^3^ or 8×10^3^ cells) in 100 μl complete medium. Cells were incubated at 37 °C in a humidified incubator with 5 % CO2 and allowed to grow for 48 or 96 h. The number of viable cells at 24, 48, 72, or 96 h was determined by using an MTS assay and absorbance at 490 nm. All experiments were performed in triplicates, and statistical significance was assessed by using Student’s t test. IL13Rα2 interleukin-13 receptor alpha 2, STAT6 signal transducer and activator of transcription 6, TP63 tumor protein p63. [file 13058_2015_607_MOESM7_ESM.pdf]

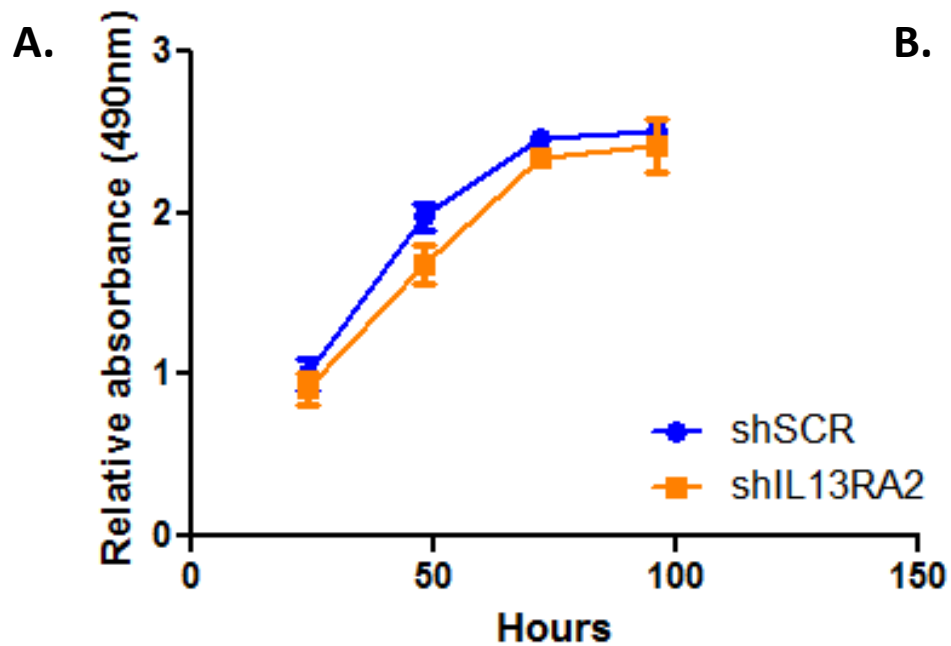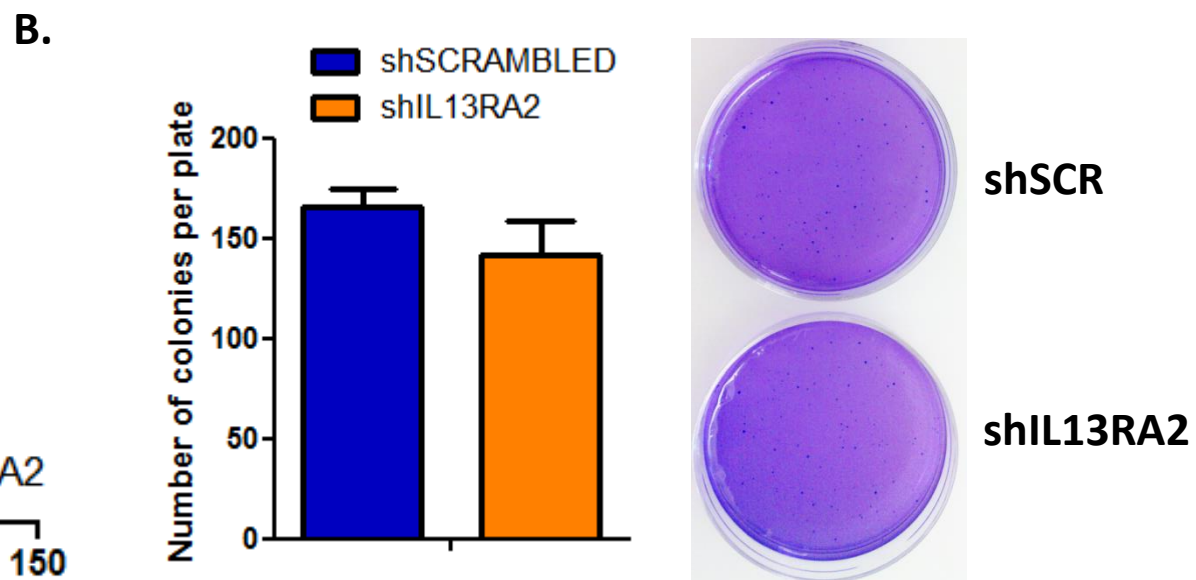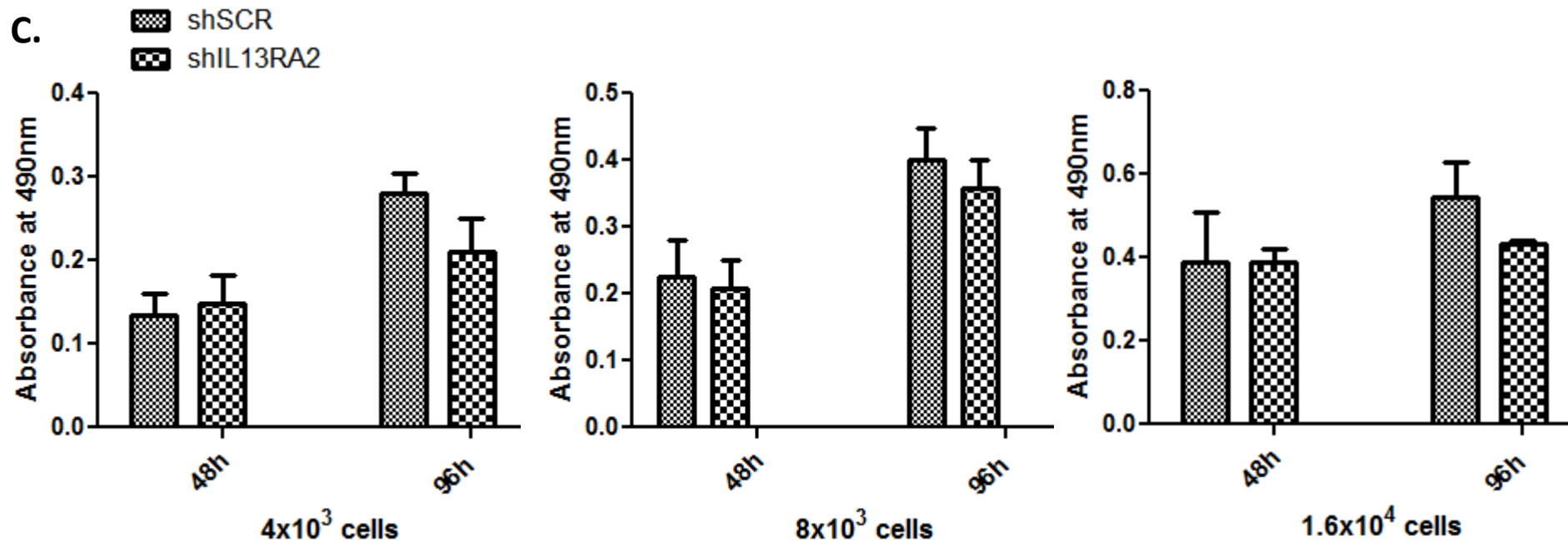

Supplement: Supplementary file 8 — IL13Rα2 silencing does not affect breast cancer cell colonization in the lungs. (a) Comparison of the number of macroscopic nodules in the right lung of non-obese diabetic/severe combined immunodeficient mice injected with 5×10^5^ MIV-Luc-shSCR or MIV-Luc-shIL13Rα2#2. Cells were inoculated directly into the right lung of mice by injection into the upper margin of the sixth intercostal rib on the right anterior axillary line, as previously described [47]. Animals (n = 4) were monitored daily over a period of up to 52 days, and each mouse was euthanized when it developed notable cachexia symptoms. When mice were sacrificed, the lungs were excised and the number of macroscopic nodules in the right lung was counted and compared between the two groups. (b) Survival curves for the two animal groups were generated on the basis of the days that mice were euthanized. Statistical significance was assessed by using Student’s t test. IL13Rα2 interleukin-13 receptor alpha 2, NS not significant, shIL13Rα2 small hairpin RNA against interleukin-13 receptor alpha 2, shSCR scrambled small hairpin RNA. [file 13058_2015_607_MOESM8_ESM.pdf]

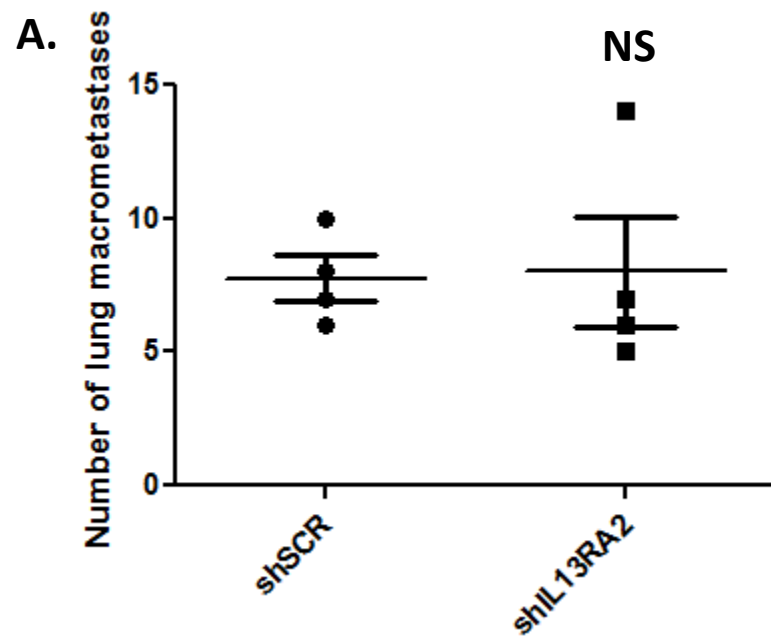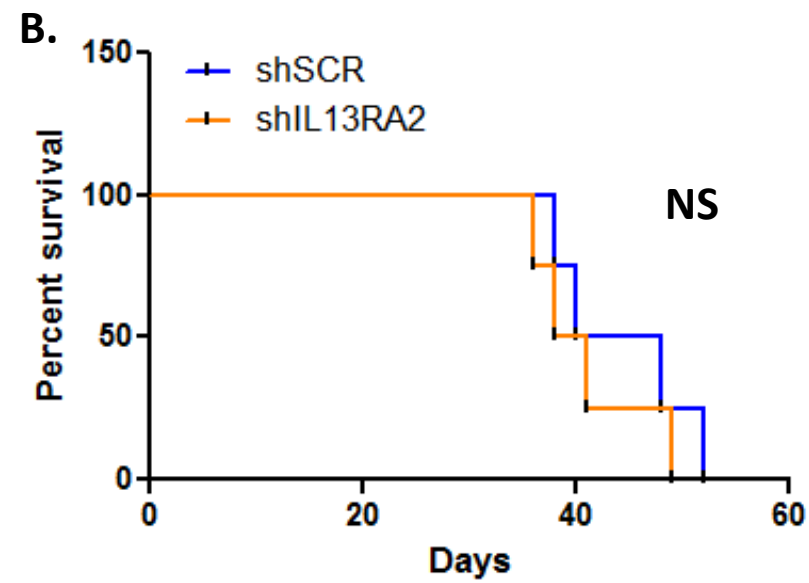

Supplement: Supplementary file 9 — IL13Rα2 depletion and IL-13 treatment additively suppress breast cancer cell migration. Representative images from transwell migration assays. Cells proficient or deficient in IL13Rα2, treated with 20 ng/ml IL-13 for 48 h or not, that were localized on the bottom membrane surface were stained with trypan blue (0.4 %) in order to quantify their migratory potential. IL-13 interleukin-13, IL13Rα2 interleukin-13 receptor alpha 2. [file 13058_2015_607_MOESM9_ESM.pdf]

## A. TP63 RFS

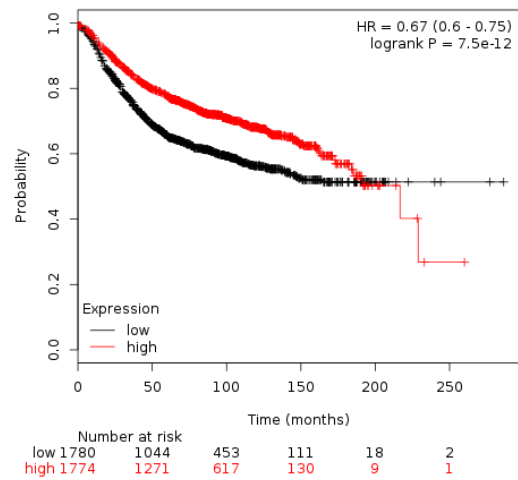

## B. TP63 DMFS

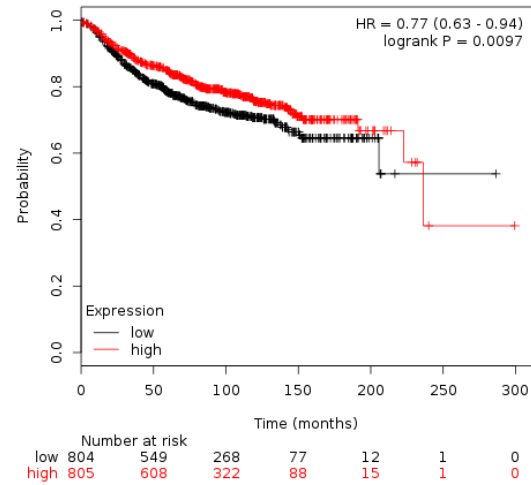

## STAT6 RFS

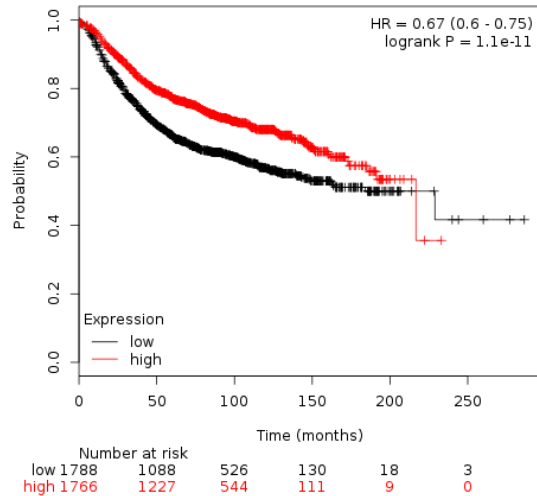

## STAT6 DMFS

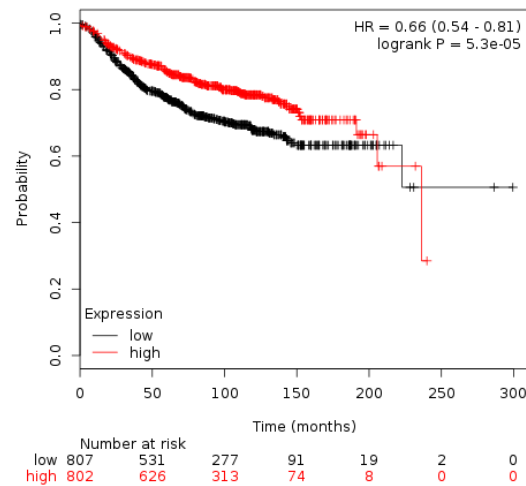

## TP63, STAT6 combined RFS

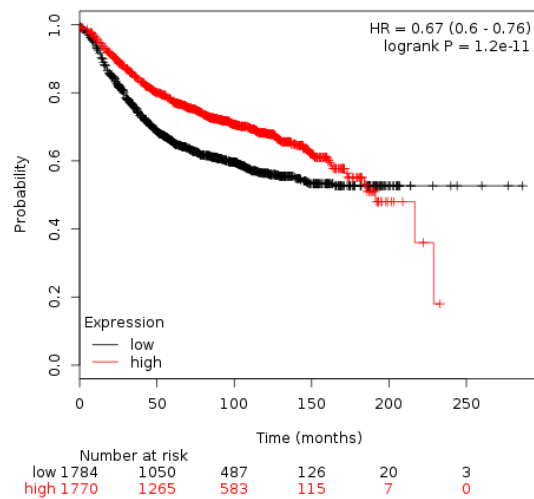

Supplement: Supplementary file 10 — Kaplan-Meier survival plot analysis for STAT6 and TP63. (a) Kaplan-Meier relapse-free survival (RFS) plots for patients with breast cancer were generated by using the Kaplan-Meier Plotter online tool [24] based on data stratified based on the median tumor expression of STAT6 and TP63 individually or based on their median expression combined. (b) Distant metastasis-free survival (DMFS) analysis of patients with breast cancer, based on the median tumor expression of STAT6 and TP63 individually. All curves were compared by log-rank test. STAT6 signal transducer and activator of transcription 6, TP63 tumor protein p63. [file 13058_2015_607_MOESM10_ESM.pdf]
