# Supplementary material for: Targeting IL13Ralpha2 activates STAT6-TP63 pathway to suppress breast cancer lung metastasis
Source: Breast Cancer Res. 2015 Jul 25;17(1):98. doi: 10.1186/s13058-015-0607-y (PMC4531803; doi:10.1186/s13058-015-0607-y)
Supplement: Supplementary file 3 — Kaplan-Meier survival plot analysis of candidate metastasis-promoting genes. Kaplan-Meier survival plots were generated by using the Kaplan-Meier Plotter online tool [24] based on data stratified at either the lower quartile or the median expression of each gene, as indicated. (a) Distant metastasis-free survival (DMFS) analysis of patients for all breast cancers was calculated on the basis of tumor IL13Rα2 expression for each of the following genes: SMTN, AGTPBP1, IGF2BP2, VEGFA, HMGCS1, TRIB3, INSIG1, TMEM97, INHBA, and IL13RA2. Curves were compared by log-rank test. (b) DMFS analysis of patients for different tumor grades (1, 2, or 3) was calculated on the basis of tumor IL13RA2 expression for each of the following genes: SMTN, AGTPBP1, IGF2BP2, VEGFA, HMGCS1, TRIB3, INSIG1, TMEM97, and INHBA. Curves were compared by log-rank test. IL13Rα2 interleukin-13 receptor alpha 2. [file 13058_2015_607_MOESM3_ESM.pdf]

A.

All breast cancers

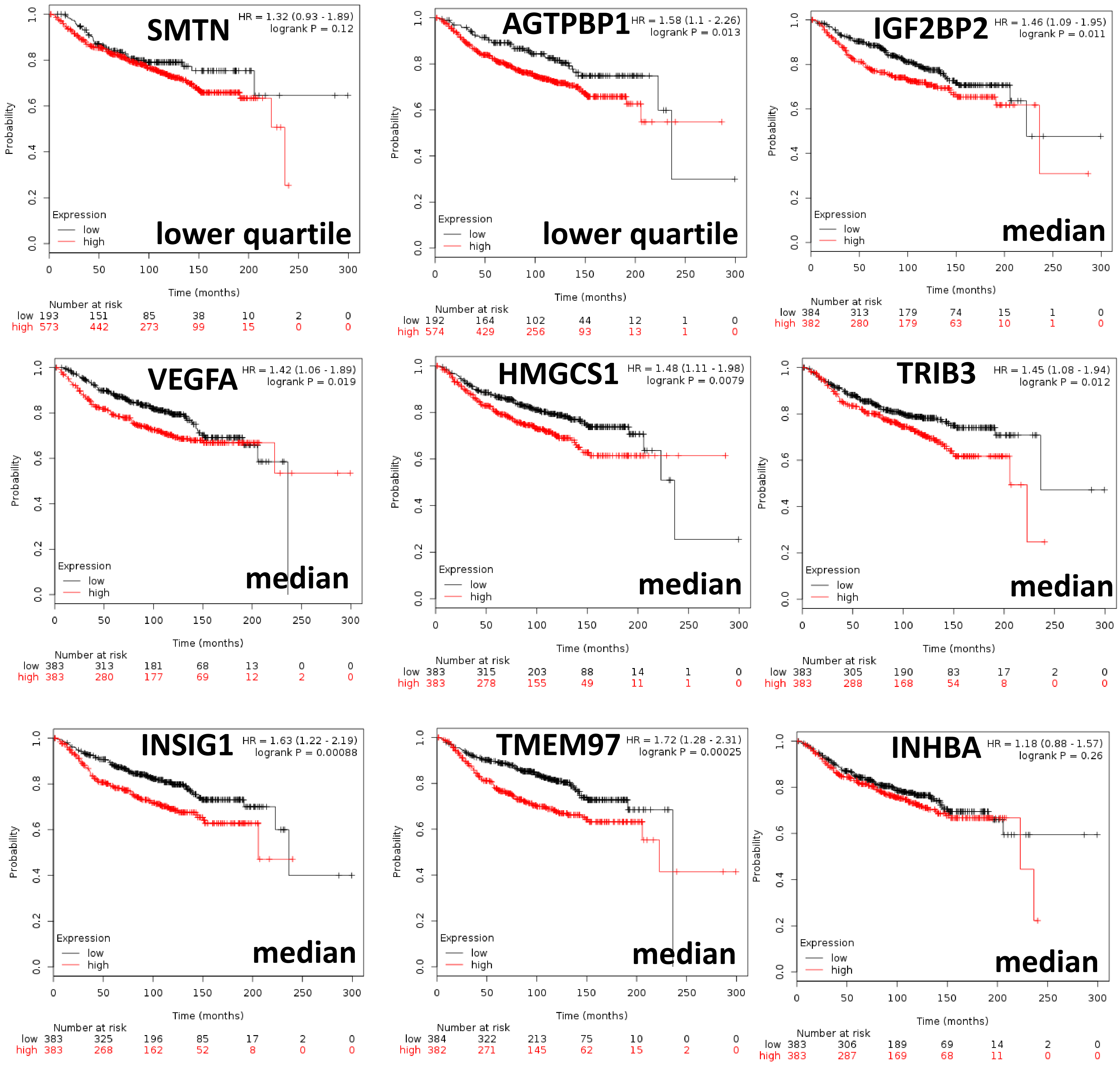

All breast cancers

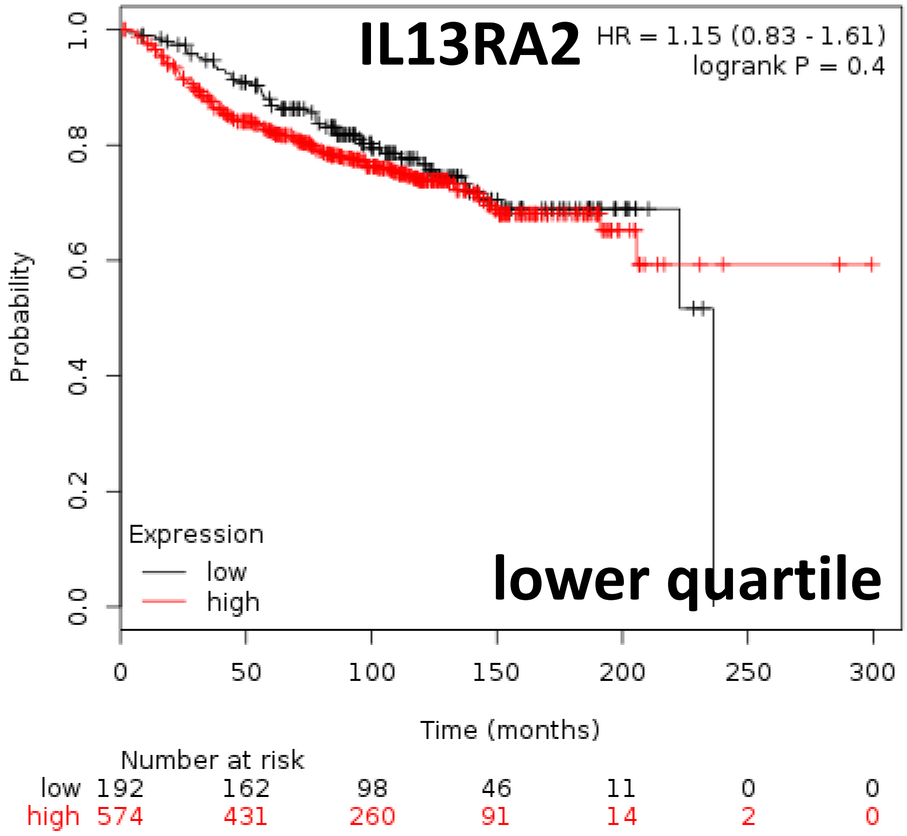

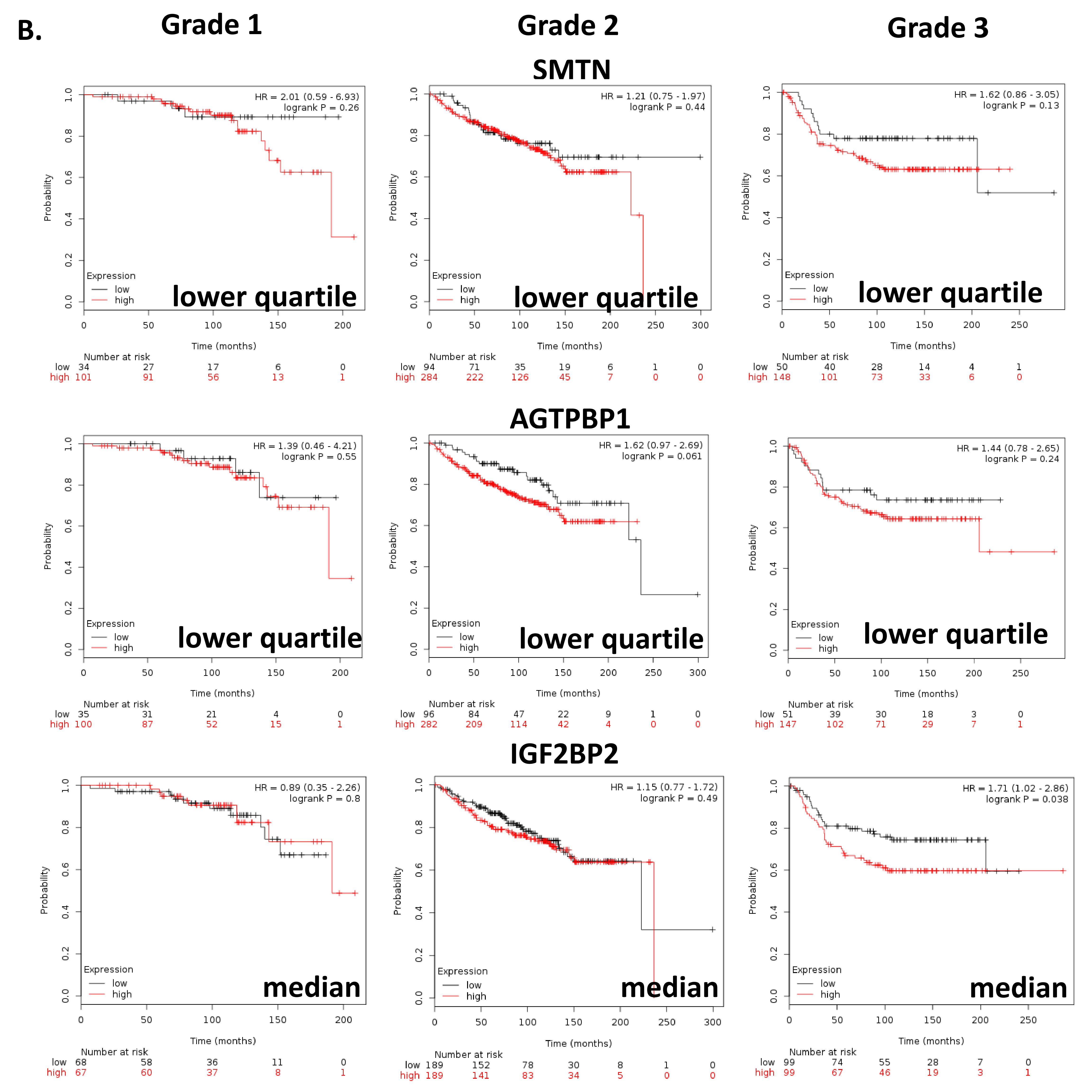

B.
Grade 1
Grade 2
Grade 3
SMTN
HR = 2.01 (0.59 - 6.93)
logrank P = 0.26
lower quartile
Number at risk
low 34 27 17 6 0
high 101 91 56 13 1
HR = 1.21 (0.75 - 1.97)
logrank P = 0.44
lower quartile
Number at risk
low 94 71 35 19 6 1 0
high 284 222 126 45 7 0 0
HR = 1.62 (0.86 - 3.05)
logrank P = 0.13
lower quartile
Number at risk
low 50 40 28 14 4 1
high 148 101 73 33 6 0
AGTPBP1
HR = 1.39 (0.46 - 4.21)
logrank P = 0.55
lower quartile
Number at risk
low 35 31 21 4 0
high 100 87 52 15 1
HR = 1.62 (0.97 - 2.69)
logrank P = 0.061
lower quartile
Number at risk
low 96 84 47 22 9 1 0
high 282 209 114 42 4 0 0
HR = 1.44 (0.78 - 2.65)
logrank P = 0.24
lower quartile
Number at risk
low 51 39 30 18 3 0
high 147 102 71 29 7 1
IGF2BP2
HR = 0.89 (0.35 - 2.26)
logrank P = 0.8
median
Number at risk
low 68 58 36 11 0
high 67 60 37 8 1
HR = 1.15 (0.77 - 1.72)
logrank P = 0.49
median
Number at risk
low 189 152 78 30 8 1 0
high 189 141 83 34 5 0 0
HR = 1.71 (1.02 - 2.86)
logrank P = 0.038
median
Number at risk
low 99 74 55 28 7 0
high 99 67 46 19 3 1
Probability
Time (months)
Expression
low
high

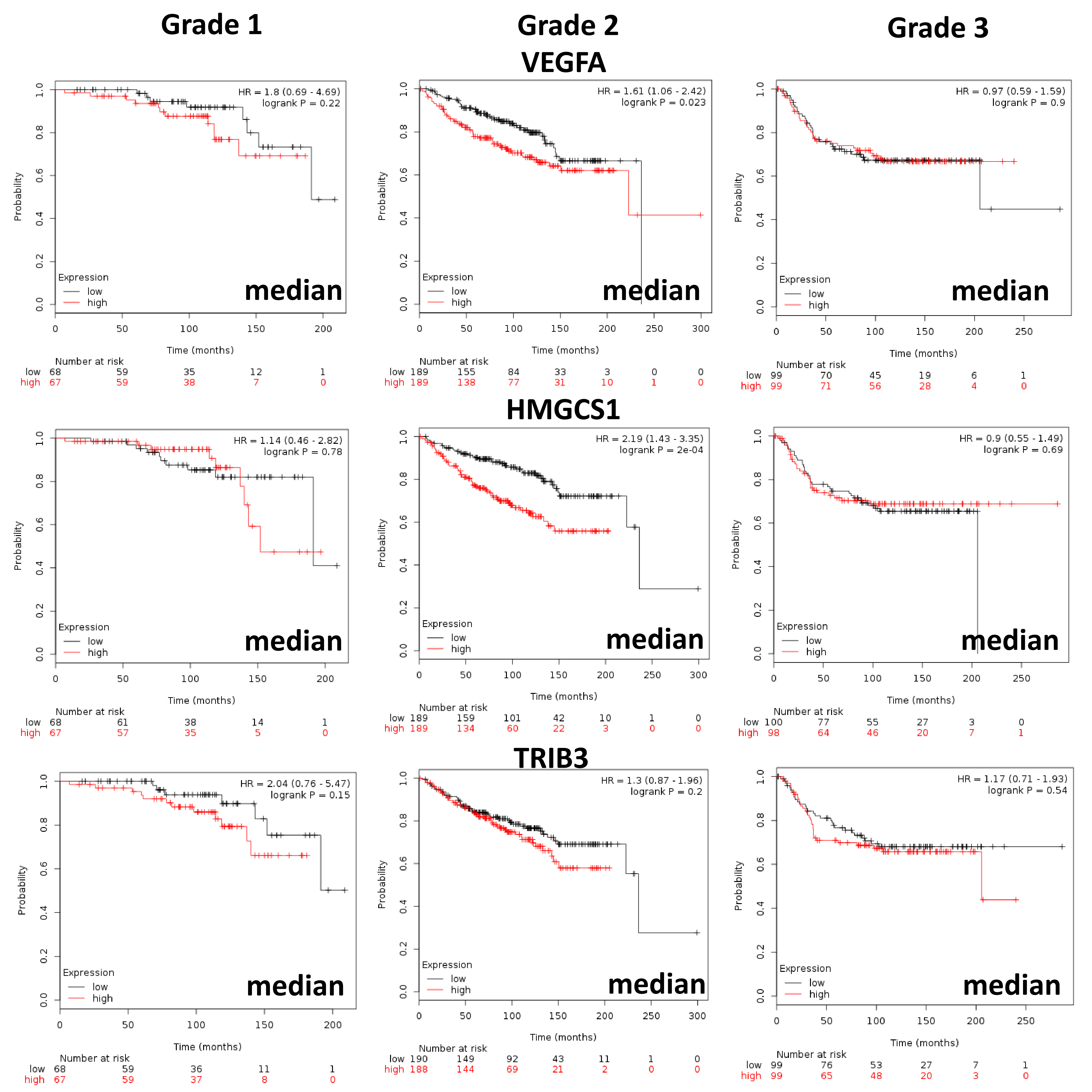
Grade 1
Grade 2
Grade 3
VEGFA
HR = 1.8 (0.69 - 4.69)
logrank P = 0.22
Expression
low
high
median
Probability
Time (months)
Number at risk
low 68 59 35 12 1
high 67 59 38 7 0
HR = 1.61 (1.06 - 2.42)
logrank P = 0.023
Expression
low
high
median
Probability
Time (months)
Number at risk
low 189 155 84 33 3 0 0
high 189 138 77 31 10 1 0
HR = 0.97 (0.59 - 1.59)
logrank P = 0.9
Expression
low
high
median
Probability
Time (months)
Number at risk
low 99 70 45 19 6 1
high 99 71 56 28 4 0
HMGCS1
HR = 1.14 (0.46 - 2.82)
logrank P = 0.78
Expression
low
high
median
Probability
Time (months)
Number at risk
low 68 61 38 14 1
high 67 57 35 5 0
HR = 2.19 (1.43 - 3.35)
logrank P = 2e-04
Expression
low
high
median
Probability
Time (months)
Number at risk
low 189 159 101 42 10 1 0
high 189 134 60 22 3 0 0
HR = 0.9 (0.55 - 1.49)
logrank P = 0.69
Expression
low
high
median
Probability
Time (months)
Number at risk
low 100 77 55 27 3 0
high 98 64 46 20 7 1
TRIB3
HR = 2.04 (0.76 - 5.47)
logrank P = 0.15
Expression
low
high
median
Probability
Time (months)
Number at risk
low 68 59 36 11 1
high 67 59 37 8 0
HR = 1.3 (0.87 - 1.96)
logrank P = 0.2
Expression
low
high
median
Probability
Time (months)
Number at risk
low 190 149 92 43 11 1 0
high 188 144 69 21 2 0 0
HR = 1.17 (0.71 - 1.93)
logrank P = 0.54
Expression
low
high
median
Probability
Time (months)
Number at risk
low 99 76 53 27 7 1
high 99 65 48 20 3 0

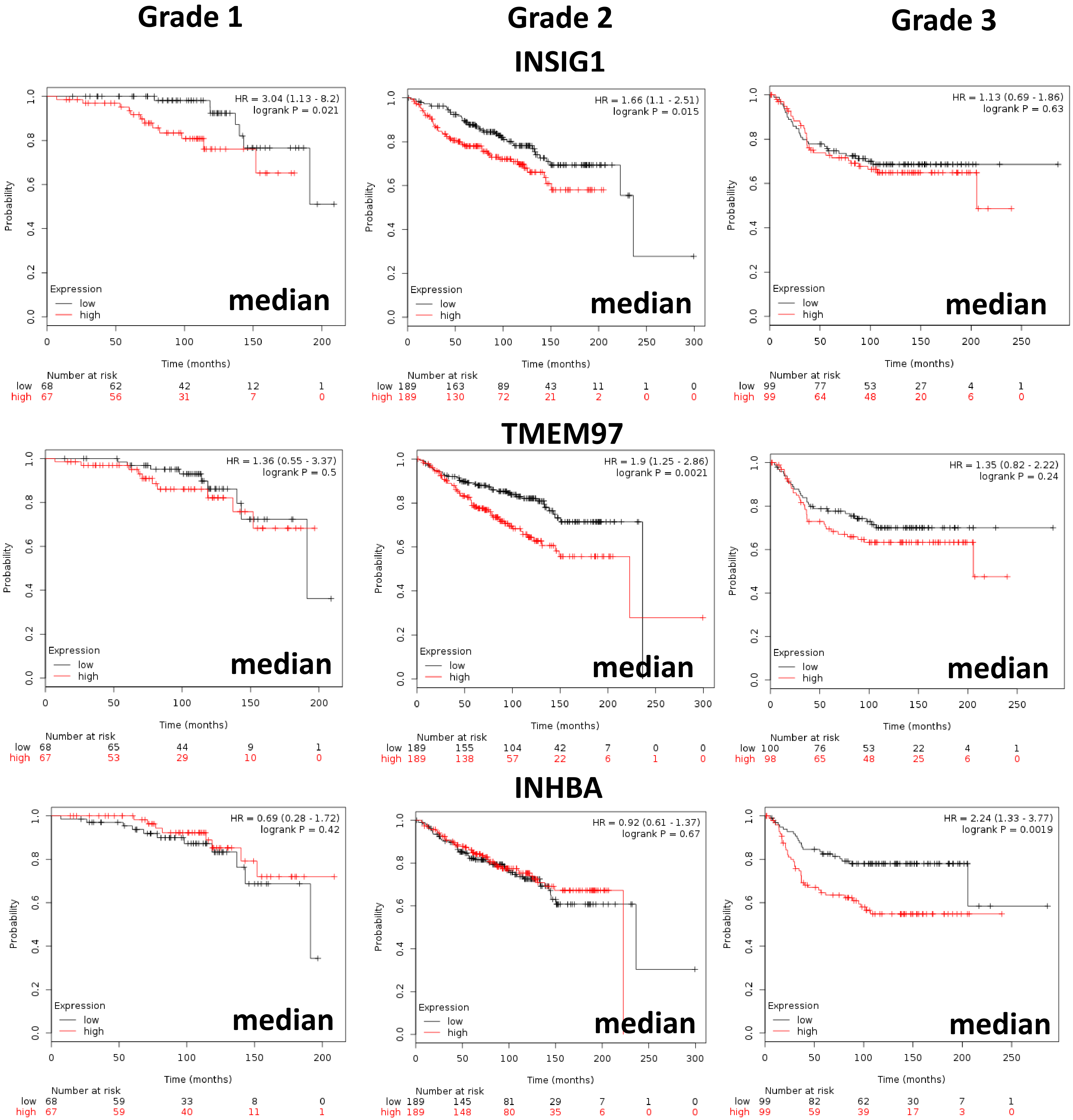

Grade 1
Grade 2
Grade 3
INSIG1
HR = 3.04 (1.13 - 8.2)
logrank P = 0.021
Expression
low
high
median
Probability
Time (months)
Number at risk
low 68 62 42 12 1
high 67 56 31 7 0
HR = 1.66 (1.1 - 2.51)
logrank P = 0.015
Expression
low
high
median
Probability
Time (months)
Number at risk
low 189 163 89 43 11 1 0
high 189 130 72 21 2 0 0
HR = 1.13 (0.69 - 1.86)
logrank P = 0.63
Expression
low
high
median
Probability
Time (months)
Number at risk
low 99 77 53 27 4 1
high 99 64 48 20 6 0
TMEM97
HR = 1.36 (0.55 - 3.37)
logrank P = 0.5
Expression
low
high
median
Probability
Time (months)
Number at risk
low 68 65 44 9 1
high 67 53 29 10 0
HR = 1.9 (1.25 - 2.86)
logrank P = 0.0021
Expression
low
high
median
Probability
Time (months)
Number at risk
low 189 155 104 42 7 0 0
high 189 138 57 22 6 1 0
HR = 1.35 (0.82 - 2.22)
logrank P = 0.24
Expression
low
high
median
Probability
Time (months)
Number at risk
low 100 76 53 22 4 1
high 98 65 48 25 6 0
INHBA
HR = 0.69 (0.28 - 1.72)
logrank P = 0.42
Expression
low
high
median
Probability
Time (months)
Number at risk
low 68 59 33 8 0
high 67 59 40 11 1
HR = 0.92 (0.61 - 1.37)
logrank P = 0.67
Expression
low
high
median
Probability
Time (months)
Number at risk
low 189 145 81 29 7 1 0
high 189 148 80 35 6 0 0
HR = 2.24 (1.33 - 3.77)
logrank P = 0.0019
Expression
low
high
median
Probability
Time (months)
Number at risk
low 99 82 62 30 7 1
high 99 59 39 17 3 0
