# Supplementary material for: Targeting IL13Ralpha2 activates STAT6-TP63 pathway to suppress breast cancer lung metastasis
Source: Breast Cancer Res. 2015 Jul 25;17(1):98. doi: 10.1186/s13058-015-0607-y (PMC4531803; doi:10.1186/s13058-015-0607-y)
Supplement: Supplementary file 11 — List of differentially expressed genes between MIV-shSCR (−IL13) VS MIV-shSCR (+IL13) cells. shSCRscrambled small hairpin RNA. [file 13058_2015_607_MOESM11_ESM.pdf]

Suppl. Table 1: **shSCR (-IL13) VS shSCR (+IL13)**

Upregulated	Fold change	Downregulated	Fold change
SERPINB4	25.18886131	CASP14	-2.717061
SERPINB3	14.73262907	AGR2	-2.357287
TNFAIP6	3.646856671	TMEM45A	-2.295109
CCL26	3.236737797	RNU4-2	-2.128151
PDCD1LG2	3.20739603	LOC285456	-2.110632
SIDT1	2.776661865	VTCN1	-2.104689
CA2	2.754480024	CEACAM6	-2.049327
SLC28A3	2.701375138	LOC100134868	-2.003656
ABCC2	2.623712242		
FAP	2.597126885		
GCNT3	2.594683376		
LRAT	2.523911809		
SERPINB13	2.441599378		
IL13RA2	2.406963893		
SLIT2	2.213663601		
SLC26A2	2.210708796		
TP63	2.128176049		
DPYD	2.080073489		
RNU5B-1	2.031664244		
DNASE2B	2.017610935		
