# Supplementary material for: Targeting IL13Ralpha2 activates STAT6-TP63 pathway to suppress breast cancer lung metastasis
Source: Breast Cancer Res. 2015 Jul 25;17(1):98. doi: 10.1186/s13058-015-0607-y (PMC4531803; doi:10.1186/s13058-015-0607-y)
Supplement: Supplementary file 12 — List of differentially expressed genes between MIV-shSCR (−IL13) VS MIV-shIL13RA2 (−IL13) cells. shIL13Rα2 small hairpin RNA against interleukin-13 receptor alpha 2, shSCR scrambled small hairpin RNA. [file 13058_2015_607_MOESM12_ESM.pdf]

Suppl. Table 2: shSCR (-IL13) VS shIL13RA2 (-IL13)

Upregulated	Fold change	Downregulated	Fold change
CASP14	5.960467539	IL13RA2	-3.13395
SULT1E1	3.536197861	RNU4-2	-2.476196
GPX2	3.093075395	CXCL17	-2.175239
TMPRSS15	3.042368697	PSPH	-2.166544
CEACAM5	3.007237894	SERPINA1	-2.160342
LOC644714	2.532040534	ZNF487P	-2.143314
MLLT3	2.430252979	ARL14	-2.006565
PLCB1	2.426222734		
LOC400986	2.426124355		
BICC1	2.409466095		
LPHN3	2.3955742		
CLCA2	2.385970987		
CDH19	2.363531349		
C3orf57	2.33407513		
ADAD2	2.31116298		
TP63	2.283371338		
SULT1B1	2.257993761		
MAP2	2.253182232		
ANKRD11	2.252798846		
GAGE12J	2.20802652		
ZNF711	2.165771441		
RASA4	2.144327852		
HPGD	2.13836629		
RXFP1	2.090209428		
COL12A1	2.046721065		
GCRG224	2.040495459		
TAS2R4	2.026289065		
SERPINB4	2.007797819		
MME	2.006515777		
